# N-Donor ligand activation of titanocene for the Biginelli reaction *via* the imine mechanism†

**DOI:** 10.1039/c8ra01208c

**Published:** 2018-02-27

**Authors:** Shaohua Zheng, Yajun Jian, Shan Xu, Ya Wu, Huaming Sun, Guofang Zhang, Weiqiang Zhang, Ziwei Gao

**Affiliations:** Key Laboratory of Applied Surface and Colloid Chemistry, MOE, School of Chemistry and Chemical Engineering, Shaanxi Normal University Xi’an 710062 China yajunjian@snnu.edu.cn zwgao@snnu.edu.cn +86-29-81530727; College of Chemistry and Chemical Engineering, Xi’an Shiyou University Xi’an 710065 P. R. China

## Abstract

The remarkable activation of stable titanocene dichloride (Cp_2_TiCl_2_) was achieved using N-donor ligand urea in an alcoholic solvent, leading to the formation of a Ti(iv) species [(MeO)_2_Ti(NHCONH_2_)]^+^, the existence of which was verified by ESI-MS, ESI-MS/MS, and NMR. Catalyzed by the newly formed Ti(iv) species, a myriad of 3,4-dihydropyrimidin-2-(1*H*)-ones were produced *via* a three-component Biginelli reaction. Further mechanistic investigation indicated that the Biginelli reaction had taken place *via* the imine route.

## Abstract 2

Stable titanocene dichloride (Cp_2_TiCl_2_) was activated by the N-donor ligand urea to form [(MeO)_2_Ti(NHCONH_2_)]^+^, which catalyzed the Biginelli reaction to produce 3,4-dihydropyrimidin-2-(1*H*)-ones.

## Graphical abstract

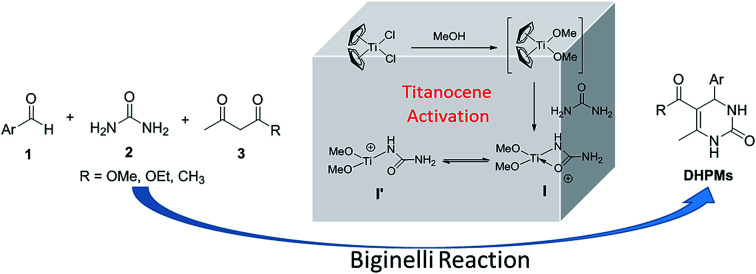

The Biginelli reaction, discovered in 1891 by Italian chemist Pietro Biginelli, is an acid-catalyzed three-component reaction of ethyl acetoacetate, aldehyde, and urea to afford 3,4-dihydropyrimidin-2-(1*H*)-ones (DHPMs).^1^ In the past few decades, this old-fashioned MCR has experienced a remarkable revival, especially due to the pharmacological and therapeutic properties of DHPMs and their derivatives,^2^ such as their antiviral, antitumor, antibacterial, and anti-inflammatory activity.^3–6^ Owing to this considerable attention towards DHPMs, a wide range of novel protocols has been developed in the last two decades, notably featuring the usage of Lewis acids as catalysts, such as LiClO_4_,^7^ LaCl_3_,^8^ InCl_3_,^9^ Yb(OTf)_3_,^10^ Cu(OTf)_2_,^11^*etc.* In addition, some sustainable conditions have also been reported, including the adoption of ionic liquids,^12^ or microwave irradiation.^13^ More important contributions are the attempted investigations into the mechanism: Folkers and Johnson^14^ proposed that the reaction was initiated by the adduction of urea with benzaldehyde or ethyl acetate; De Souza^15^ captured several intermediates by ESI-(+)-MS for the proposal of the iminium mechanism under their reaction conditions; Neto^16^ found excess reagents are required for the transformation of the precatalyst CuCl_2_ into the active catalytic species; Neto^17^ also concluded that the iminium mechanism is the preferred pathway based on a systematic kinetic pathway investigation; Neto^18^ found that the catalyst not only improves the yield but is also responsible for the selection of the preferred reaction pathway; Sherwood^19^ elucidated that the combination of the catalyst and the solvent elevated the reaction productivity. All these experimental and mechanistic studies provide great inspiration in the search for a novel, efficient procedure for the Biginelli reaction.

Although the currently-used Lewis acid-based catalysts for the Biginelli reaction are milder catalysts, their acidity and catalytic ability are difficult to modulate, because most are inorganic salts.^7–11^ One of the effective strategies is to use stable organometallic complexes as pre-catalysts, with their electronic features modulated by facile coordination or solvation. Our group has long been involved in modulating the Lewis acidity of group IVB metallocenes like Cp_2_TiCl_2_.^20–22^ Until now, the successful activation strategies for Cp_2_TiCl_2_ primarily involve O-donor or P-donor ligands to form more active species, such as Cp_2_Ti[P(OEt)_3_]_2_,^23^ [Ti(Cp)_2_(H_2_O)_2_(CF_3_SO_3_)_2_],^24^ and [Ti(Cp)_2_(H_2_O)_2_(C_8_F_17_SO_3_)_2_].^25^ Through the *in situ* establishment of those active titanocene-based Lewis acids, many organic transformations like the Mannich^26–28^ and Friedel–Crafts reactions^29^ have been realized with satisfying yields (Scheme 1). The reason that Ti(iv)–N bond-containing complexes are rare can be partially attributed to the weakness of Ti(iv)–N bonding compared with Ti(iv)–O bonding,^30^ although weaker Ti(iv)–N bonding may feature stronger catalytic activity. Herein, we present the N-donor ligand activation of the pre-catalyst Cp_2_TiCl_2_ to form a novel Ti(iv) species [(MeO)_2_Ti(NHCONH_2_)]^+^ for the Biginelli reaction for the synthesis of a myriad of dihydropyrimidinone derivatives in an alcoholic solvent and in satisfying yield. This systematic mechanistic study shows that the reaction takes place *via* the imine route.

**Scheme 1:**
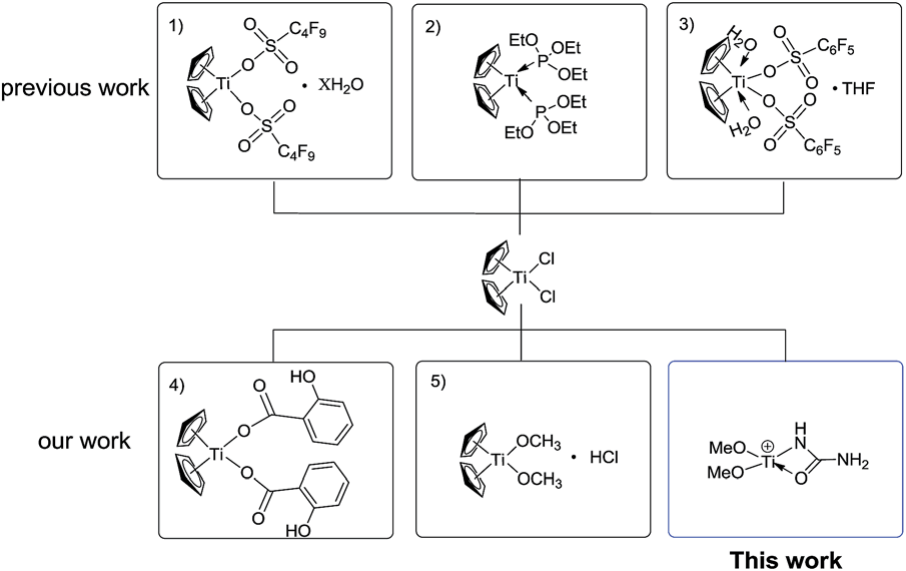
Selected activated Cp_2_TiCl_2_ as Lewis acids.

To start with, the Biginelli reaction using aldehyde 1, urea 2, ethyl acetoacetate 3, and Cp_2_TiCl_2_ was chosen as the model reaction. Our previous work^22^ indicated that a methanol solvent could activate the inert Cp_2_TiCl_2_ by forming a new titanocene species Cp_2_Ti(OMe)_2_ and the Brønsted acid HCl, leading to successful C–C/C–N bond formation. Therefore, the solvent effect was tested initially, and it was not surprising that the yield in alcoholic MeOH and EtOH was superior to that in other solvents, which is in great accordance with our previous findings. With the optimal solvent EtOH in hand, we further studied how the temperature affected the reaction efficiency. As shown in Table 1, 70 °C was the best reaction temperature, at which an 85% (entry 7) yield of the final product was generated, and either lower temperature (60 °C, entry 6) or higher temperature (80 °C, entry 8) produced much less product. Subsequently, we systematically examined the relationship between catalyst loading and the reaction yield, and, considering longer reaction times lead to a higher yield, the reaction times were all extended to 9 h. As is shown in Table 1 (entry 9–13), the catalyst plays a very important role in the reaction; only trace product was generated in the absence of Cp_2_TiCl_2_, and the yield increased when the catalyst loading was elevated, but with a descending rate, with the yield reaching 99% upon 15 mol% catalyst loading. Taking the catalyst loading and catalytic efficiency together into account, a 10 mol% amount was chosen as the best catalyst amount to be used. To sum up, the optimal reaction conditions were as displayed in entry 11, where the Biginelli reaction took place in EtOH at 70 °C, with 10 mol% Cp_2_TiCl_2_, leading to a 93% yield of DHPM.

**Table 1:** Optimization of the Cp_2_TiCl_2_ promoted Biginelli reaction^a^^,^^b^

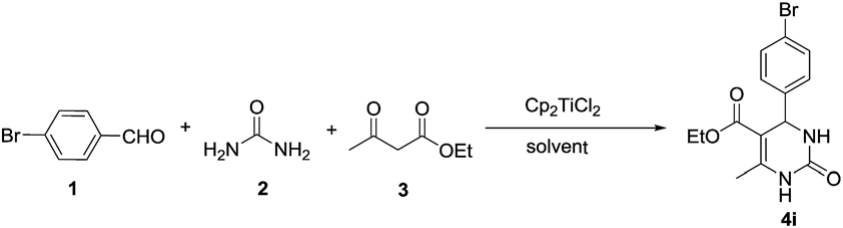					
Entry	Solvent	Temp. (°C)	Catalyst loading (%)	Time (h)	Yield (%)
1	MeOH	60	10	5	38
2	Hexane	60	10	5	28
3	Toluene	60	10	5	8
4	DMSO	60	10	5	8
5	MeCN	60	10	5	24
6	EtOH	60	10	5	43
7	EtOH	70	10	5	85
8	EtOH	80	10	5	60
9	EtOH	70	0	24	Trace
10	EtOH	70	5	9	82
11	EtOH	70	10	9	93
12	EtOH	70	15	9	99
13	EtOH	70	20	9	99

a Reaction conditions: a mixture of *p*-bromo benzaldehyde 1 (1 mmol), urea 2 (2 mmol), and ethyl acetoacetate 3 (1 mmol) in the presence of Cp_2_TiCl_2_ for a period time as needed.

b Isolated yield.

To demonstrate the utility of this method, two components – the aldehyde and the 1,3-diketone – were varied subsequently. We initially examined various substituted aromatic aldehydes (Scheme 2). The results showed that aromatic aldehydes bearing electron-donating groups (Scheme 2, 4b–4f) gave DHPMs in high yields of more than 82%. The exception is when –OMe is at the *m*-position with respect to the –CHO group; the yield decreased to only 67%, and we tentatively attributed such abnormity to the electron-donating behavior from the oxygen atom’s lone electron pair to the –CHO group through p–π conjugation, which attenuated the reaction activity of the aldehyde. The yield of those aldehydes with electron-withdrawing substituent groups was severely related to the substitution position with respect to the –CHO group as well. For the aldehydes where the *para*-H was replaced by electron-withdrawing groups, DHPM yields of at least 81% could be produced (Scheme 2, 4g–4i), whereas when *o*,*m*-H was replaced, the corresponding DHPMs were obtained only in moderate yields from 67% to 78% (Scheme 2, 4j–4m). The limitations of our newly developed protocol were the alkyl aldehydes, where the yields dropped significantly to 12% and 50% (4n, 4o, respectively).

**Scheme 2:**
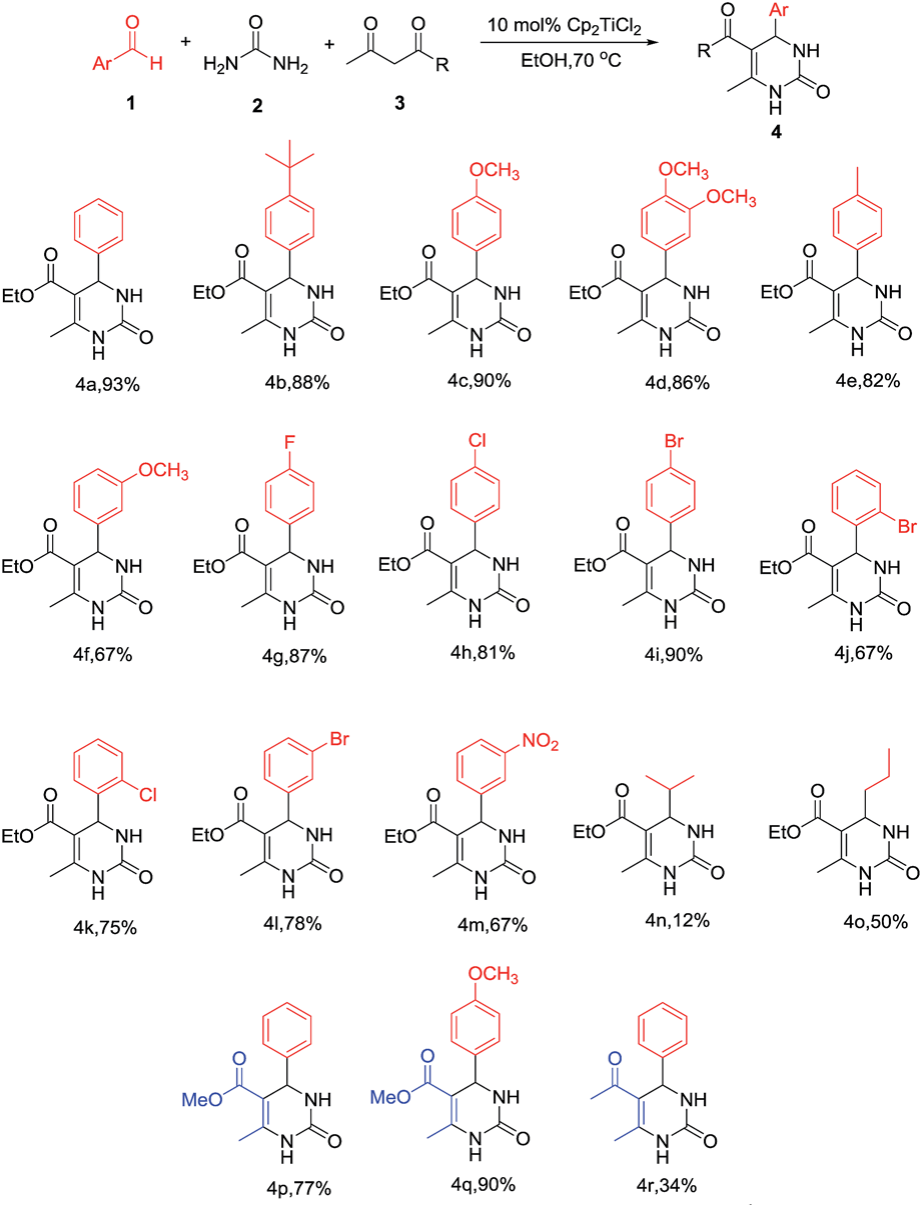
Substrate scope for the synthesis of DHPMs^*a,b*^. ^*a*^Reaction conditions: a mixture of benzaldehyde 1 (1 mmol), urea 2 (2 mmol), ethyl acetoacetate 3 (1 mmol) and Cp_2_TiCl_2_ (0.1 mmol) was stirred in EtOH at 70 °C for 9 h. ^*b*^Isolated yield obtained after purification by column chromatography.

To shed light on how inert Cp_2_TiCl_2_ promotes the Biginelli reaction, it is of great importance and indispensability to figure out its conversion to its active species. As shown in Scheme 1, Cp_2_TiCl_2_ is usually activated by O-donor ligands, whereas their incubation with benzaldehyde or ethyl acetoacetate leads to no changes (corresponding NMR data can be seen in the ESI†). To our surprise, when it came to the incubation of Cp_2_TiCl_2_ with urea in ethanol, the characteristic orange color of Cp_2_TiCl_2_ disappeared, suggesting some changes had happened. As shown in Fig. 1a, all the NMR signals correspond to Cp_2_TiCl_2_ and undeuterated ethanol, indicating ethanol cannot activate Cp_2_TiCl_2_ by itself. In apparent contrast, when extra urea was added, the Cp signal disappeared in less than 15 min, as monitored by *in situ*^13^C NMR, and the occurrence of three new peaks with their chemical shifts at 133.57, 132.50, and 41.71 ppm suggested the production of cyclopentadiene. In order to dig deeper, ESI-MS experiments were conducted in methanol; the reason for the adoption of methanol as the reaction solvent was because the MS signal is more sensitive than in ethanol. The new primary titanium ion of 169.0087 *m*/*z* might correspond to [(MeO)_2_Ti(NHCONH_2_)]^+^, further solidifying the rupture of Cp–Ti bonding. Taking the NMR and MS data together, we propose the conversion route for Cp_2_TiCl_2_ (Fig. 1c): under the cooperation of basic urea, Cp_2_TiCl_2_ transformed into Cp_2_Ti(OMe)_2_, which further reacted with urea to form intermediate I, whereupon the Cp–Ti bond breaking happened. As the reaction happened in an alcoholic solvent, an equilibrium between I and I′ may exist, and I might be the true catalysis species.

**Fig. 1:**
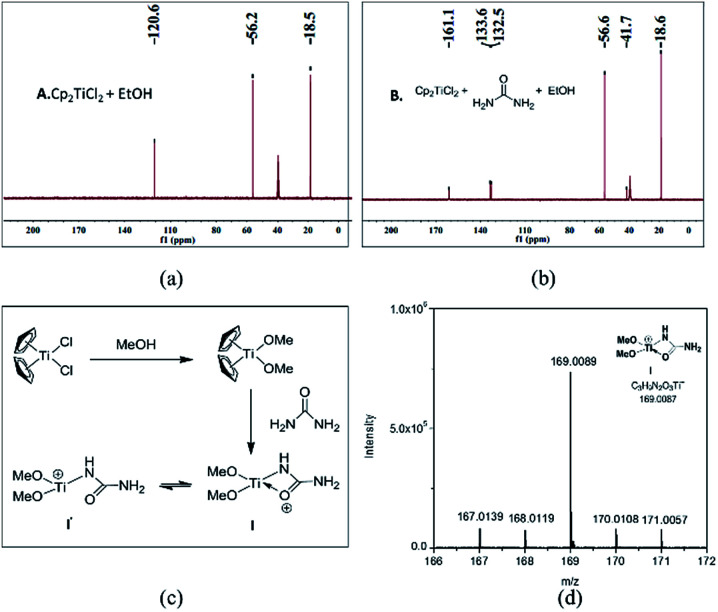
(a) ^13^C NMR of Cp_2_TiCl_2_ in the presence of EtOH (DMSO-*d*_6_); (b) ^13^C NMR of Cp_2_TiCl_2_ in the presence of EtOH and urea (DMSO-*d*_6_); (c) transformation pathway of Cp_2_TiCl_2_ in the presence of urea in an alcoholic solvent; (d) key intermediate I captured by ESI-(+)-MS.

Given that the catalytic species was found, we then focused on mapping the reaction route of the reaction. Generally, the three-component Biginelli reaction initiates from the condensation of two components, so it is important to figure out how the first condensation happens. Theoretically, there are three possible combinations^14,31,32^ that can be expected: (1) Cp_2_TiCl_2_, aldehyde, and urea, (2) Cp_2_TiCl_2_, ethyl acetoacetate, and urea, or (3) Cp_2_TiCl_2_, ethyl acetoacetate, and aldehyde. Therefore, three paralleled experiments were conducted to check the formation rate of imine, enamine, and Knoevenagel adducts. As shown in Scheme 3, the imine (a) and enamine (b) could be generated in 86% and 24% yield, respectively, in 1 h, whereas no Knoevenagel adduct could be detected, leading to the conclusion that the Cp_2_TiCl_2_ catalyzed Biginelli reaction was initiated starting from the imine route (a) or enamine route (b).

**Scheme 3:**
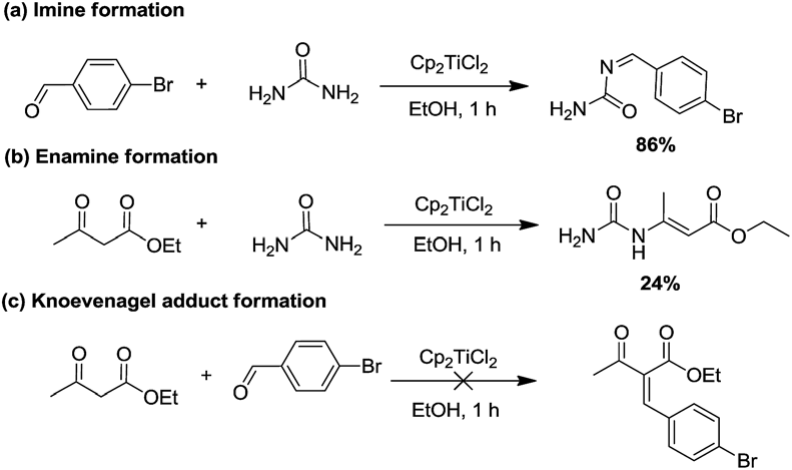
The formation of (a) imine, (b) enamine, and (c) Knoevenagel adducts promoted by Cp_2_TiCl_2_ in EtOH.

To further elucidate if the present Cp_2_TiCl_2_ promoted Biginelli reaction had taken place through these two routes, we further investigated whether the enamine route (path b) is also a minor possible pathway or just a condensation product produced during the reaction. Enamine was produced *via* mixing urea (1 equiv.) and ethyl acetoacetate (1 equiv.) with Cp_2_TiCl_2_ (10 mol%), and the resulting enamine product was incubated with aldehyde and extra urea to see if DHPMs could be generated. Negative results suggested that enamine adduct formation is just an end product, but not an intermediate to the Biginelli products *via* the enamine route (Scheme 4).

**Scheme 4:**
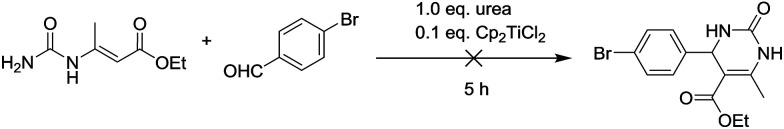
Reaction of enamine, aldehyde, and urea promoted by Cp_2_TiCl_2_.

From the above mechanistic analysis, two points can be proposed: (1) the true catalysis species is [(MeO)_2_Ti(NHCONH_2_)]^+^, and (2) the Biginelli reaction took place through the imine route. Because previous findings were obtained based on controlled experiments with the absence of some reactants, we carried out the full Biginelli reaction, monitored by ESI-MS at certain time intervals, and a series of Ti species were detected (Fig. 2A). The detection of the primary signal at 169.0089 *m*/*z* further consolidated the role of intermediate I as the catalytic species, while the ion of 334.9520 *m*/*z* corresponds to the species where the ligand replacement of urea by the imine formed intermediate II. The signal at the *m*/*z* value of 465.0136 indicated that the Ti center was coordinated by both ethyl acetoacetate and the imine adduct to form intermediate III. It is worthwhile to mention here that the existence of I is helpful for the activation of ethyl acetate upon the formation of VI – corresponding to the signal at 239.0393 *m*/*z* – with the sharp comparison that nothing happens between Cp_2_TiCl_2_ and ethyl acetoacetate without the involvement of urea. To further verify the proposed structure for these intermediates, ESI-(+)-MS/MS spectrometry was also conducted. As shown in Fig. 2B, the fragmentation of I results in two major peaks at *m*/*z* values of 151.9798, and 126.0014, which correspond to the loss of NH_3_ and NH_2_–C=O, respectively. For the fragmentation process of intermediate VI, two peaks at *m*/*z* values of 211.0088, and 192.9975 were seen, corresponding to the loss of CH_2_=CH_2_, and further loss of a H_2_O (Fig. 2C). As shown in Fig. 2D, the fragmentation of III results in the major peak at a *m*/*z* value of 239.0410, corresponding to the loss of the imine adduct between urea and aldehyde 1.

**Fig. 2:**
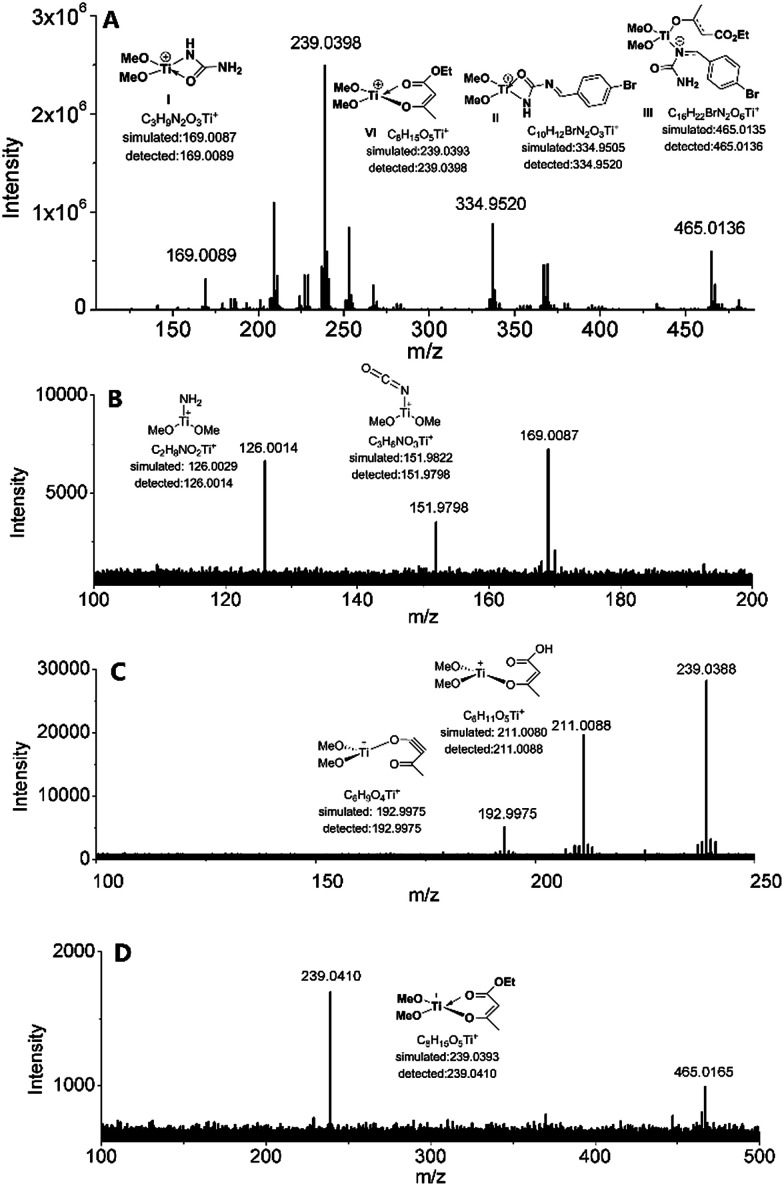
(A) High resolution ESI-(+)-MS of a reaction mixture of *p*-bromo benzaldehyde 1 (1 mmol), urea 2 (2 mmol), ethyl acetoacetate 3 (1 mmol), and Cp_2_TiCl_2_ (0.1 mmol) at 70 °C for 2 h; (B) ESI-(+)-MS/MS spectrum of intermediate I; (C) ESI-(+)-MS/MS spectrum of intermediate VI; (D) ESI-(+)-MS/MS spectrum of intermediate III.

Considering the NMR, ESI-MS, ESI-MS/MS, and parallel experiments together, a plausible catalytic cycle was proposed (Scheme 5). At the beginning, inert Cp_2_TiCl_2_ is converted into Cp_2_Ti(OMe)_2_, followed by urea ligand replacement to form quadrivalent titanium I [(MeO)_2_Ti(NHCONH_2_)]^+^, which acts as the authentic catalytic species. Once I is generated, the free NH_2_ group will further react with benzaldehyde to produce intermediate II, which can coordinate with another ethyl acetoacetate at the titanium metal center to generate intermediate III. As is shown in the catalytic cycle, the C–C bond formation between the imine and enol parts of III to give intermediate IV is facilitated by a titanium involving six-membered ring transition state. Upon the formation of IV, an extra urea molecule coordinates with the Ti center to regenerate catalyst I and release compound V, and then intramolecular condensation between NH_2_ and the ketone, and tautomerization leads to the formation of the target DHPM. Besides this imine route, another enamine route (path B) is also involved, but fails to produce the target molecule, where the amino group on catalytic species I can react with ethyl acetoacetate to form VII, which might not be active enough to react with benzaldehyde to finish the catalytic cycle to produce DHPM.

**Scheme 5:**
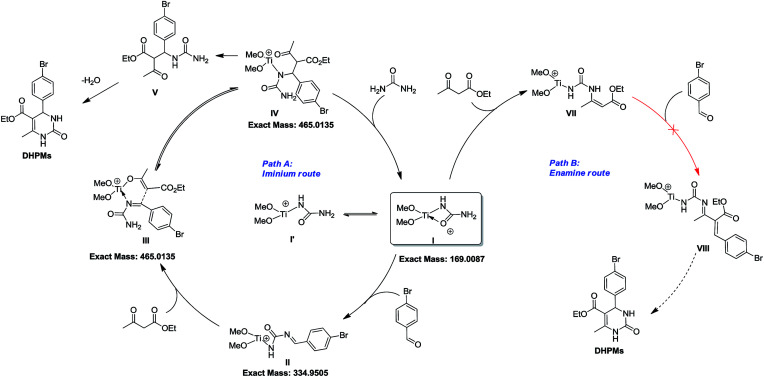
Proposed mechanism for the synthesis of DHPMs catalyzed by novel Ti(iv) species.

In summary, we have developed a novel N-donor ligand activation strategy for inert Cp_2_TiCl_2_, where the Lewis acidity of titanium is tuned by urea in an alcoholic solvent to form [(MeO)_2_Ti(NHCONH_2_)]^+^. The newly formed Ti(iv) species catalyzes the Biginelli reaction with high efficiency, generating a series of 3,4-dihydropyrimidin-2-(1*H*)-ones under mild conditions. Thorough mechanistic study *via in situ* NMR and MS indicates that the imine route contributes to the formation of the Biginelli product, and the enamine route is terminated on the way to the final product, at the point where the enamine condensation product is produced. Further investigation and development of other N-donor ligands is underway in our laboratory.

## Conflicts of interest

There are no conflicts to declare.

## Supplementary Material

RA-008-C8RA01208C-s001

## References

[cit1] Biginelli P. (1893). Gazz. Chim. Ital..

[cit2] Kappe C. O. (2000). Acc. Chem. Res..

[cit3] Suresh, Sandhu J. S. (2012). ARKIVOC.

[cit4] Ashok M., Holla B. S., Kumari N. S. (2007). Eur. J. Med. Chem..

[cit5] Prashantha Kumar B. R., Sankar G., Nasir Baig R. B., Chandrashekaran S. (2009). Eur. J. Med. Chem..

[cit6] Kappe C. O. (1998). Molecules.

[cit7] Yadav J. S., Reddy B. V. S., Srinivas R., Venugopal C., Ramalingam T. (2001). Synthesis.

[cit8] Khabazzadeh H., Saidi K., Sheibani H. (2008). Bioorg. Med. Chem. Lett..

[cit9] Ranu B. C., Hajra A., Jana U. (2000). J. Org. Chem..

[cit10] Ma Y., Qian C., Wang L., Yang M. (2000). J. Org. Chem..

[cit11] Paraskar A. S., Dewkar G. K., Sudalai A. (2003). Tetrahedron Lett..

[cit12] Javidi J., Esmaeilpour M., Dodeji F. N. (2015). RSC Adv..

[cit13] Pasunooti K. K., Chai H., Jensen C. N., Gorityala B. K., Wang S. M., Liu X. W. (2011). Tetrahedron Lett..

[cit14] Folkers K., Johnson T. B. (1933). J. Am. Chem. Soc..

[cit15] De Souza R. O., da Penha E. T., Milagre H. M., Garden S. J., Esteves P. M., Eberlin M. N., Antunes O. A. (2009). Chem.–Eur. J..

[cit16] Ramos L. M., Ponce de Leon y Tobio A. Y., dos Santos M. R., de Oliveira H. C., Gomes A. F., Gozzo F. C., de Oliveira A. L., Neto B. A. (2012). J. Org. Chem..

[cit17] Ramos L. M., Guido B. C., Nobrega C. C., Correa J. R., Silva R. G., de Oliveira H. C., Gomes A. F., Gozzo F. C., Neto B. A. (2013). Chem.–Eur. J..

[cit18] Alvim H. G., Lima T. B., de Oliveira A. L., de Oliveira H. C., Silva F. M., Gozzo F. C., Souza R. Y., da Silva W. A., Neto B. A. (2014). J. Org. Chem..

[cit19] Clark J. H., Macquarrie D. J., Sherwood J. (2013). Chem.–Eur. J..

[cit20] Chapman A. M., Wass D. F. (2012). Dalton Trans..

[cit21] Sloan M. E., Staubitz A., Clark T. J., Russell C. A., Lloyd-Jones G. C., Manners I. (2010). J. Am. Chem. Soc..

[cit22] Saito T., Nishiyama H., Tanahashi H., Kawakita K., Tsurugi H., Mashima K. (2014). J. Am. Chem. Soc..

[cit23] Takeda T., Ando M., Sugita T., Tsubouchi A. (2007). Org. Lett..

[cit24] Hollis T. K., Robinson N. P., Bosnich B. (1992). Organometallics.

[cit25] Qiu R., Xu X., Peng L., Zhao Y., Li N., Yin S. (2012). Chem.–Eur. J..

[cit26] Wu Y., Chen C., Jia G., Zhu X., Sun H., Zhang G., Zhang W., Gao Z. (2014). Chem.–Eur. J..

[cit27] Zhu X., Chen C., Yu B., Zhang G., Zhang W., Gao Z. (2014). Chem. Lett..

[cit28] Wu Y., Wang X., Luo Y. L., Wang J., Jian Y. J., Sun H. M., Zhang G. F., Zhang W. Q., Gao Z. W. (2016). RSC Adv..

[cit29] Wang X., Wang Z., Zhang G., Zhang W., Wu Y., Gao Z. (2016). Eur. J. Org. Chem..

[cit30] Casarin M., Vittadini A., Schubert U. (2007). Monatsh. Chem..

[cit31] Sweet F., Fissekis J. D. (1973). J. Am. Chem. Soc..

[cit32] Kappe C. O. (1997). J. Org. Chem..

