# Supplementary material for: N-Donor ligand activation of titanocene for the Biginelli reaction via the imine mechanism
Source: RSC Adv. 2018 Feb 27;8(16):8657–61. doi: 10.1039/c8ra01208c (PMC9078600; doi:10.1039/c8ra01208c)
Supplement: RA-008-C8RA01208C-s001 [file RA-008-C8RA01208C-s001.pdf]

Electronic Supplementary Material (ESI) for RSC Advances.
This journal is © The Royal Society of Chemistry 2018

***Supporting Information***

Key Laboratory of Applied Surface and Colloid Chemistry (Ministry of Education), School of Chemistry and Chemical Engineering, Shaanxi Normal University, Xi'an 710062, P. R. China
E-mail: zwgao@snnu.edu.cn; Fax: +86-029-81530821

Table of Context

**1. General Information** ........................................2

**2. General Procedure for Synthesis of 3,4-dihydropyrimidin-2(1H)-ones** ..............3

**3. Experimental and Characterization Data** ........................................4

**4. $^{1}$H and $^{13}$C Spectra for 3,4-dihydropyrimidin-2(1H)-ones Products** ...................9

**5. HR-ESI-MS studies for proposed mechanism.** ........................................27

**6. $^{13}$C NMR Spectra studies for proposed mechanism** ........................................28

## 1. General Information

Reagents were purchased from commercial sources and were used as received unless mentioned otherwise. Reactions were monitored by thin layer chromatography using silica gel.$^{1}H$ and $^{13}C$ NMR spectra were recorded on a Bruker EQUINX55 (400 MHz for $^{1}H$; 101 MHz for $^{13}C$) spectrometer by using DMSO-$d_6$ as a solvent. For $^{1}H$ NMR, tetramethylsilane (TMS) served as internal standard ($\delta$ = 0) and $^{1}H$ NMR chemical shifts are reported in ppm downfield of tetramethylsilane and referenced to residual solvent peak (DMSO-$d_6$ at 2.5 ppm and 3.33 ppm) unless otherwise noted. The data are reported as follows: chemical shift, integration, multiplicity (s = singlet, d = doublet, t = triplet, q = quartet and m = multiplet), and coupling constant in Hz. For $^{13}C$ NMR, DMSO-$d_6$ was used as internal standard ($\delta$ =39.52) and spectra were obtained with complete proton decoupling.

ESI-MS and ESI-MS/MS measurements were performed in the positive-ion mode (m/z 50−2500 range) on an MAXIS instrument from Bruker. This instrument has a hybrid quadrupole/ion mobility/orthogonal acceleration time-of-flight (oa-TOF) geometry and was used in the TOF V+ mode. All samples were dissolved in methanol and were directly infused into the ESI source at a flow rate of 4.0L/min after 1 min at 180 °C. ESI source conditions were as follows: capillary voltage 4.0 kV, nebulizer 0.4 bar, scan begin 100m/z, scan end 1300m/z, collision cell RF 200.0 Vpp, end plate offset -500V.

## 2. General Procedure for Synthesis of 3,4-dihydropyrimidin-2(1H)-ones

A representative example for preparation of 4i is as following: a mixture of p-Bromo Benzaldehyde (185 g, 1 mmol), 1,3-dicarbonyl compound (128 μL ,1 mmol), urea (0.120 g, 2 mmol), and $Cp_2TiCl_2$ (0.0248 g, 10 mol % to all of the reactants) was charged into a 50 mL pressure flask with a magnetic stirring bar. EtOH (4 mL) was subsequent added by syringe. Then the reaction system was placed in an oil-bath (70 °C) with magnetic stirring. After completion of the reaction, as indicated by TLC analysis, the reaction mixture was carried out via Silica gel flask column chromatography (eluent: petroleum ether: EtOAc = 1:1) to give the desired product 4i 304.2 mg as white solid. Yield: 90%.

**3. Experimental and Characterization Data**

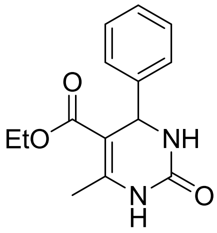

**5-Ethoxycarbonyl-6-methyl-4-phenyl-3,4-dihydropyrimidin-2(1H)-one(4a,93%)**

$^{1}$H NMR (400 MHz, DMSO) δ 9.19 (s, 1H), 7.74 (s, 1H), 7.34 – 7.29 (m, 2H), 7.24 (d, *J* = 6.9 Hz, 3H), 5.15 (d, *J* = 3.1 Hz, 1H), 3.98 (q, *J* = 7.1 Hz, 2H), 2.25 (s, 3H), 1.09 (t, *J* = 7.1 Hz, 3H). $^{13}$C NMR (101 MHz, DMSO) δ 165.4, 152.3, 148.4, 144.9, 128.4, 127.3, 126.3, 99.4, 59.3, 54.1, 17.8, 14.1.

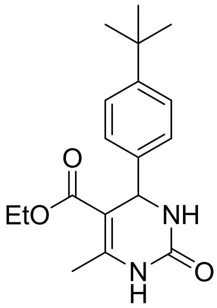

**4-(4-tert-butyl)-5-ethoxycarbonyl-6-methyl-3,4-dihydropyrimidin-2(1H)-one(4b,88%)**

$^{1}$H NMR (400 MHz, DMSO) δ 9.18 (s, 1H), 7.70 (s, 1H), 7.33 (d, *J* = 8.3 Hz, 2H), 7.17 (d, *J* = 7.9 Hz, 2H), 5.14 (d, 1H), 3.99 (q, *J* = 7.1 Hz, 2H), 2.25 (s, 3H), 1.25 (s, 9H), 1.11 (t, *J* = 7.1 Hz, 3H). $^{13}$C NMR (101 MHz, DMSO) δ 165.4, 152.4, 149.6, 148.2, 142.0, 125.9, 125.1, 99.5, 59.2, 53.5, 34.2, 31.1, 17.8, 14.1.

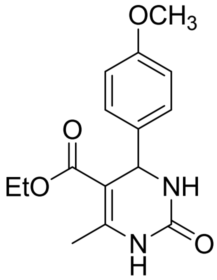

**5-Ethoxycarbonyl-4-(4-methoxyphenyl)-6-methyl-3,4-dihydropyrimidin-2(1H)-one(4c,90%)**

$^{1}$H NMR (400 MHz, DMSO) δ 9.19 (s, 1H), 7.70 (s, 1H), 7.17 (d, *J* = 8.7 Hz, 2H), 6.88 (d, *J* = 8.7 Hz, 2H), 5.13 (d, *J* = 3.2 Hz, 1H), 3.98 (q, *J* = 7.1 Hz, 2H), 3.71 (s, 3H), 2.26 (s, 3H), 1.10 (t, *J* = 7.1 Hz, 3H). $^{13}$C NMR (101 MHz, DMSO) δ 165.5, 158.6, 152.4, 148.1, 137.1, 127.5, 113.8, 99.7, 59.2, 55.1, 53.5, 17.8, 14.1.

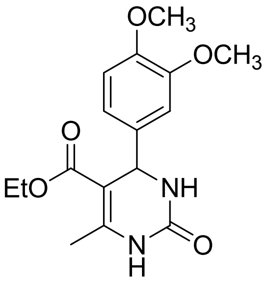

**4-(3,4-dimethoxyphenyl)-5-ethoxycarbonyl-6-methyl-3,4-dihydropyrimidin-2(1H)-one(4d,86%)**

$^{1}$H NMR (400 MHz, DMSO) δ 9.18 (s, 1H), 7.70 (s, 1H), 6.91 – 6.85 (m, 2H), 6.75 (d, *J* = 9.6 Hz, 1H), 5.13 (d, *J* = 3.0 Hz, 1H), 4.01 (q, *J* = 7.0 Hz, 2H), 3.72 (d, *J* = 3.4 Hz, 6H), 2.27 (s, 3H), 1.12 (t, *J* = 7.1 Hz, 3H). $^{13}$C NMR (101 MHz, DMSO) δ 165.5, 152.4, 148.6, 148.2 (d, *J* = 4.9 Hz), 137.4, 118.0, 111.8, 110.6, 99.5, 59.3, 55.5 (d, *J* = 10.0 Hz), 53.6, 17.8, 14.2.

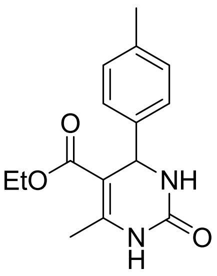

**5-Ethoxycarbonyl-4-(4-methylphenyl)-6-methyl-3,4-dihydropyrimidin-2(1H)-one(4e,82%)**

$^{1}$H NMR (400 MHz, DMSO) δ 9.16 (s, 1H), 7.69 (s, 1H), 7.12 (s, 4H), 5.12 (d, *J* = 3.2 Hz, 1H), 3.98 (q, *J* = 7.0 Hz, 2H), 2.25 (d, *J* = 5.7 Hz, 6H), 1.10 (t, *J* = 7.1 Hz, 3H). $^{13}$C NMR (101 MHz, DMSO) δ 165.4, 152.2, 148.2, 142.0, 136.4, 128.9, 126.2, 99.4, 59.2, 53.7, 20.7, 17.8, 14.1.

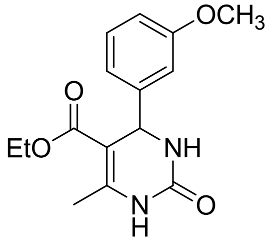

**5-Ethoxycarbonyl-4-(3-methoxyphenyl)-6-methyl-3,4-dihydropyrimidin-2(1H)-one(4f,67%)**

$^{1}$H NMR (400 MHz, DMSO) δ 9.21 (s, 1H), 7.75 (s, 1H), 7.24 (t, *J* = 7.7 Hz, 1H), 6.86 – 6.78 (m, 3H), 5.15 (d, *J* = 2.5 Hz, 1H), 4.00 (q, *J* = 7.0 Hz, 2H), 3.72 (s, 3H), 2.26 (s, 3H), 1.11 (t, *J* = 7.0 Hz, 3H). $^{13}$C NMR (101 MHz, DMSO) δ 165.4, 159.3, 152.4, 148.5, 146.4, 129.6, 118.3, 112.5, 112.2, 99.3, 59.3, 55.0, 53.9, 17.8, 14.2.

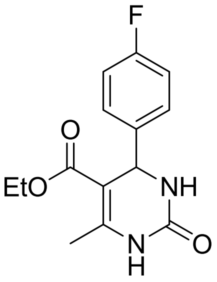

**4-(4-Fluorophenyl)-5-ethoxycarbonyl-6-methyl-3,4-dihydropyrimidin-2(1H)-one(4g,87%)**

$^{1}$H NMR (400 MHz, DMSO) δ 9.23 (s, 1H), 7.75 (s, 1H), 7.30 – 7.23 (m, 2H), 7.18 – 7.11 (m, 2H), 5.16 (d, *J* = 3.2 Hz, 1H), 4.03 – 3.93 (m, 2H), 2.26 (s, 3H), 1.09 (t, *J* = 7.1 Hz, 3H). $^{13}$C NMR (101 MHz, DMSO) δ 165.3, 162.6, 160.2, 152.1 (d, *J* = 9.3 Hz), 148.5 (d, *J* = 3.9 Hz), 141.2 (d, *J* = 3.0 Hz), 128.3 (d, *J* = 8.2 Hz), 115.1 (d, *J* = 21.1 Hz), 115.0 – 114.7 (m), 99.2 (d, *J* = 4.3 Hz), 59.2, 53.4, 17.8, 14.0.

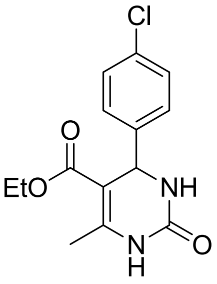

**4-(4-Chlorophenyl)-5-ethoxycarbonyl-6-methyl-3,4-dihydropyrimidin-2(1H)-one(4h,81%)**

$^{1}$H NMR (400 MHz, DMSO) δ 9.29 (s, 1H), 7.81 (s, 1H), 7.37 (d, *J* = 8.3 Hz, 2H), 7.28 (d, *J* = 8.3 Hz, 2H), 5.19 (d, *J* = 2.4 Hz, 1H), 3.98 (dd, *J* = 12.4, 6.5 Hz, 2H), 2.28 (s, 3H), 1.08 (t, *J* = 7.0 Hz, 3H).$^{13}$C NMR (101 MHz, DMSO) δ 165.3, 152.2, 148.8, 143.9, 132.0, 128.4 (d, *J* = 14.7 Hz), 99.0, 59.4, 53.6, 17.9, 14.1.

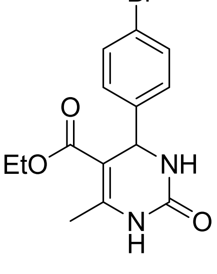

**4-(4-Bromophenyl)-5-ethoxycarbonyl-6-methyl-3,4-dihydropyrimidin-2(1H)-one(4i,90%)**

$^{1}H$ NMR (400 MHz, DMSO) δ 9.30 (s, 1H), 7.81 (s, 1H), 7.51 (d, *J* = 8.0 Hz, 2H), 7.23 (d, *J* = 8.2 Hz, 2H), 5.17 (d, *J* = 12.4 Hz, 1H), 3.98 (dd, *J* = 13.2, 6.4 Hz, 2H), 2.28 (s, 3H), 1.08 (t, *J* = 6.9 Hz, 3H). $^{13}C$ NMR (101 MHz, DMSO) δ 165.3, 152.2, 148.8, 144.3, 131.4, 128.7, 120.5, 99.0, 59.4, 53.7, 17.9, 14.1.

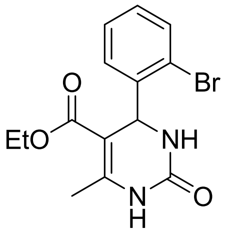


**4-(2-Bromophenyl)-5-ethoxycarbonyl-6-methyl-3,4-dihydropyrimidin-2(1H)-one(4j,67%)**

$^{1}H$ NMR (400 MHz, DMSO) δ 9.32 (s, 1H), 7.74 (s, 1H), 7.32 (dt, *J* = 28.2, 8.3 Hz, 4H), 5.67 (d, 1H), 3.90 (q, *J* = 7.1 Hz, 2H), 2.32 (s, 3H), 0.99 (t, *J* = 7.1 Hz, 3H). $^{13}C$ NMR (101 MHz, DMSO) δ 165.0, 151.6, 149.3, 141.8, 131.8, 129.4, 129.1, 128.9, 127.8, 98.0, 59.2, 51.6, 17.7, 13.9.

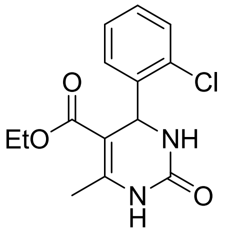


**4-(2-Chlorophenyl)-5-ethoxycarbonyl-6-methyl-3,4-dihydropyrimidin-2(1H)-one(4k,75%)**

$^{1}H$ NMR (400 MHz, DMSO) δ 9.33 (s, 1H), 7.72 (s, 1H), 7.56 (d, *J* = 7.9 Hz, 1H), 7.33 (s, 2H), 7.17 (s, 1H), 5.65 (d, *J* = 2.0 Hz, 1H), 3.90 (q, *J* = 7.0 Hz, 2H), 2.33 (s, 3H), 0.99 (t, *J* = 7.1 Hz, 3H). $^{13}C$ NMR (101 MHz, DMSO) δ 165.0, 151.5, 149.3, 143.4, 132.7, 129.4, 128.8, 128.5, 122.4, 98.5, 59.1, 54.1, 17.7, 14.0.

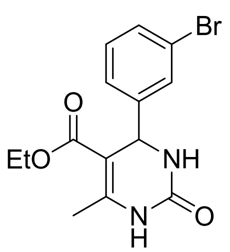


**4-(3-Bromophenyl)-5-ethoxycarbonyl-6-methyl-3,4-dihydropyrimidin-2(1H)-one(4l,78%)**

$^{1}H$ NMR (400 MHz, DMSO) δ 9.29 (s, 1H), 7.81 (s, 1H), 7.47 – 7.39 (m, 2H), 7.28 (q, *J* = 7.7 Hz, 2H), 5.18 (d, *J* = 2.9 Hz, 1H), 4.01 (dd, *J* = 14.9, 7.3 Hz, 2H), 2.28 (s, 3H), 1.10 (t, *J* = 7.1 Hz, 3H). $^{13}C$ NMR (101 MHz, DMSO) δ 165.2, 152.0, 149.0, 147.6, 130.8, 130.2, 129.3, 125.3, 121.6, 98.7, 59.4, 53.7, 17.9, 14.1.

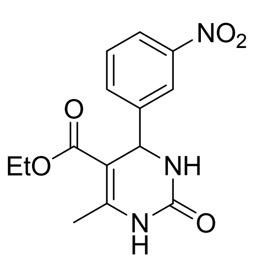


**5-Ethoxycarbonyl-6-methyl-4-(3-nitrophenyl)-3,4-dihydropyrimidin-2(1H)-one(4m,67%)**

$^{1}H$ NMR (400 MHz, DMSO) δ 9.38 (s, 1H), 8.11 (s, 2H), 7.91 (s, 1H), 7.67 (dt, *J* = 15.5, 7.7 Hz, 2H), 5.33 (d, *J* = 3.1 Hz, 1H), 3.98 (dd, *J* =

13.5, 6.7 Hz, 2H), 3.40 (s, 1H), 2.28 (s, 3H), 1.09 (t, $J$ = 7.1 Hz, 3H). $^{13}$C NMR (101 MHz, DMSO) δ 165.1, 152.0, 149.4, 147.8, 147.1, 133.1, 130.2, 122.4, 121.1, 98.5, 59.5, 53.7, 17.9, 14.0.

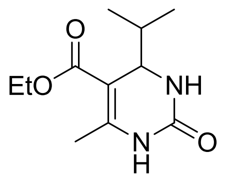


**5-Ethoxycarbonyl-6-methyl-4-isopropyl-3,4-dihydropyrimidin-2(1H)-one(4n,12%)**

$^{1}$H NMR (400 MHz, DMSO) δ 8.89 (s, 1H), 7.28 (s, 1H), 4.11 – 4.01 (m, 2H), 3.95 (t, $J$ = 3.4 Hz, 1H), 2.17 (s, 3H), 1.18 (t, $J$ = 7.1 Hz, 4H), 0.82 (d, $J$ = 6.9 Hz, 3H), 0.74 (d, $J$ = 6.8 Hz, 3H). $^{13}$C NMR (101 MHz, DMSO) δ 165.8, 153.2, 148.5, 98.2, 59.1, 55.5, 34.6, 18.5, 17.7, 16.0, 14.2.

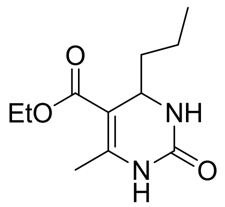


**5-Ethoxycarbonyl-6-methyl-4-propyl-3,4-dihydropyrimidin-2(1H)-one(4o,50%)**

$^{1}$H NMR (400 MHz, DMSO) δ 8.93 (s, 1H), 7.32 (s, 1H), 4.10 – 4.01 (m, 3H), 2.16 (s, 3H), 1.43 – 1.30 (m, 3H), 1.18 (t, $J$ = 7.1 Hz, 4H), 0.85 (t, $J$ = 6.9 Hz, 3H). $^{13}$C NMR (101 MHz, DMSO) δ 165.5, 152.9, 148.3, 99.5, 59.1, 49.8, 39.1, 17.7, 17.0, 14.2, 13.7.

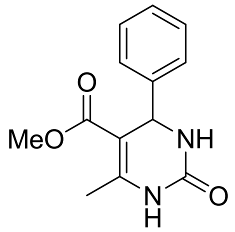


**5-Methoxycarbonyl-6-methyl-4-phenyl-3,4-dihydropyrimidin-2(1H)-one(4p,77%)**

$^{1}$H NMR (400 MHz, DMSO) δ 9.25 (s, 1H), 7.78 (s, 1H), 7.36 – 7.29 (m, 2H), 7.28 – 7.21 (m, 3H), 5.18 (d, $J$ = 3.1 Hz, 1H), 3.54 (s, 3H), 2.27 (s, 3H). $^{13}$C NMR (101 MHz, DMSO) δ 165.9, 152.3, 148.7, 144.7, 128.5, 127.3, 126.2, 99.1, 53.9, 50.8, 17.9.

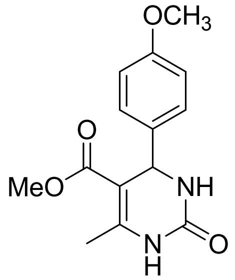


**5-Methoxycarbonyl-4-(4-methoxyphenyl)-6-methyl-3,4-dihydropyrimidin-2(1H)-one(4q,90%)**

$^{1}$H NMR (400 MHz, DMSO) δ 9.21 (s, 1H), 7.71 (s, 1H), 7.17 (d, $J$ = 8.6 Hz, 2H), 6.88 (d, $J$ = 8.6 Hz, 2H), 5.12 (d, $J$ = 3.1 Hz, 1H), 3.72 (s, 3H), 3.53 (s, 3H), 2.27 (s, 3H). $^{13}$C NMR (101 MHz, DMSO) δ 165.9, 158.5, 152.3, 148.4, 136.9, 127.4, 113.8, 99.4, 55.1, 53.3, 50.8, 17.9.

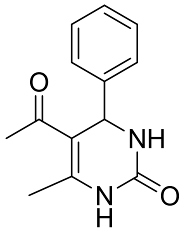


**5,6-Dimethyl-4-phenyl-3,4-dihydropyrimidin-2(1H)-one(4r,34%)**

$^{1}H$ NMR (400 MHz, DMSO) δ 9.20 (s, 1H), 7.85 (s, 1H), 7.35 – 7.31 (m, 2H), 7.26 (d, *J* = 4.8 Hz, 3H), 5.28 (d, *J* = 3.3 Hz, 1H), 2.30 (s, 3H), 2.11 (s, 3H). $^{13}C$ NMR (101 MHz, DMSO) δ 194.7, 152.6, 148.5, 144.7, 128.9, 127.8, 126.8, 110.0, 54.3, 30.7, 19.3.

## 4. $^{1}H$ and $^{13}C$ Spectra for 3,4-dihydropyrimidin-2(1H)-ones Products

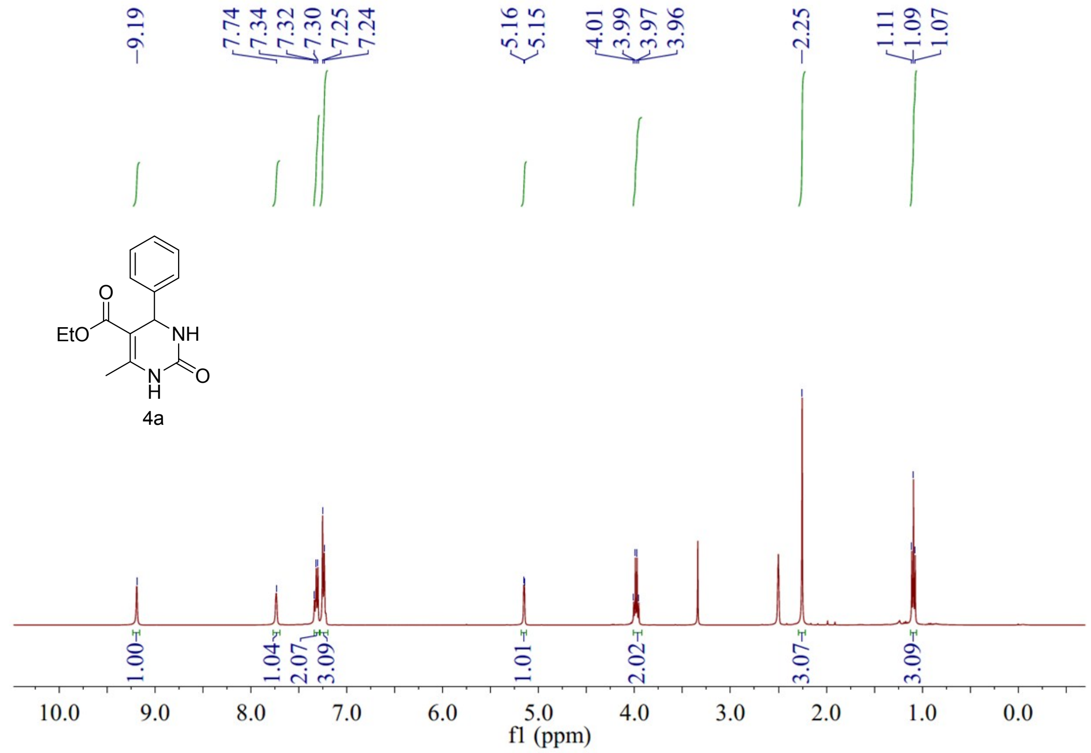


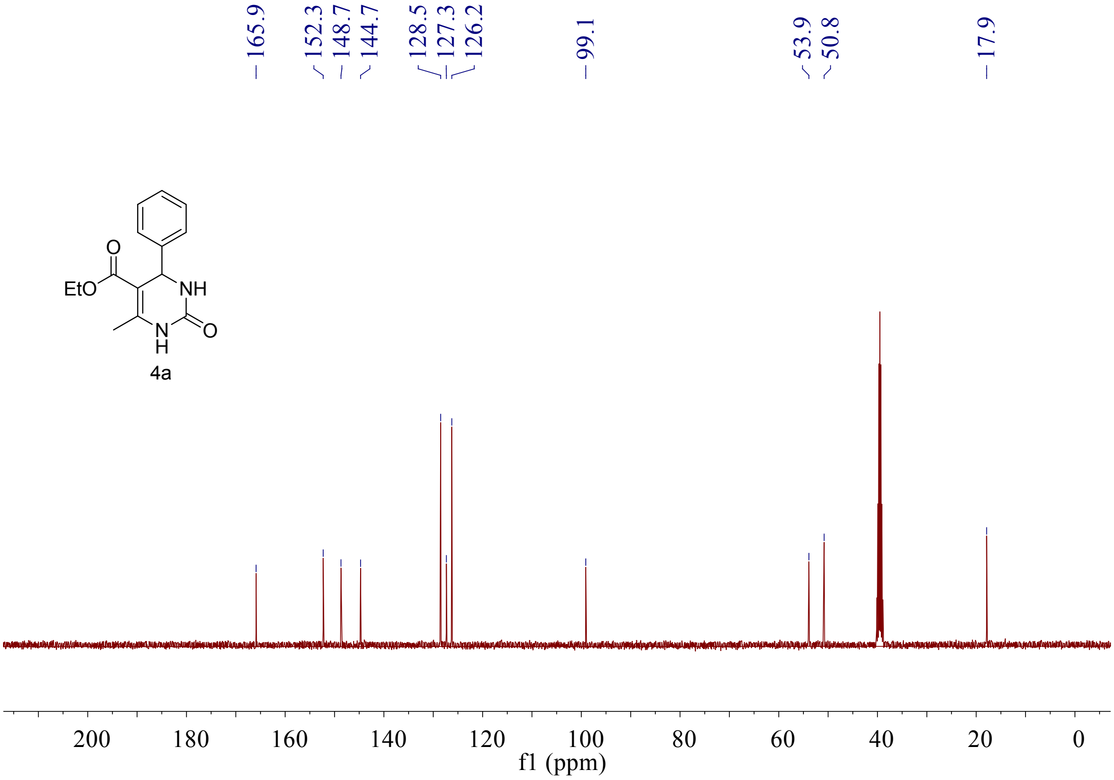

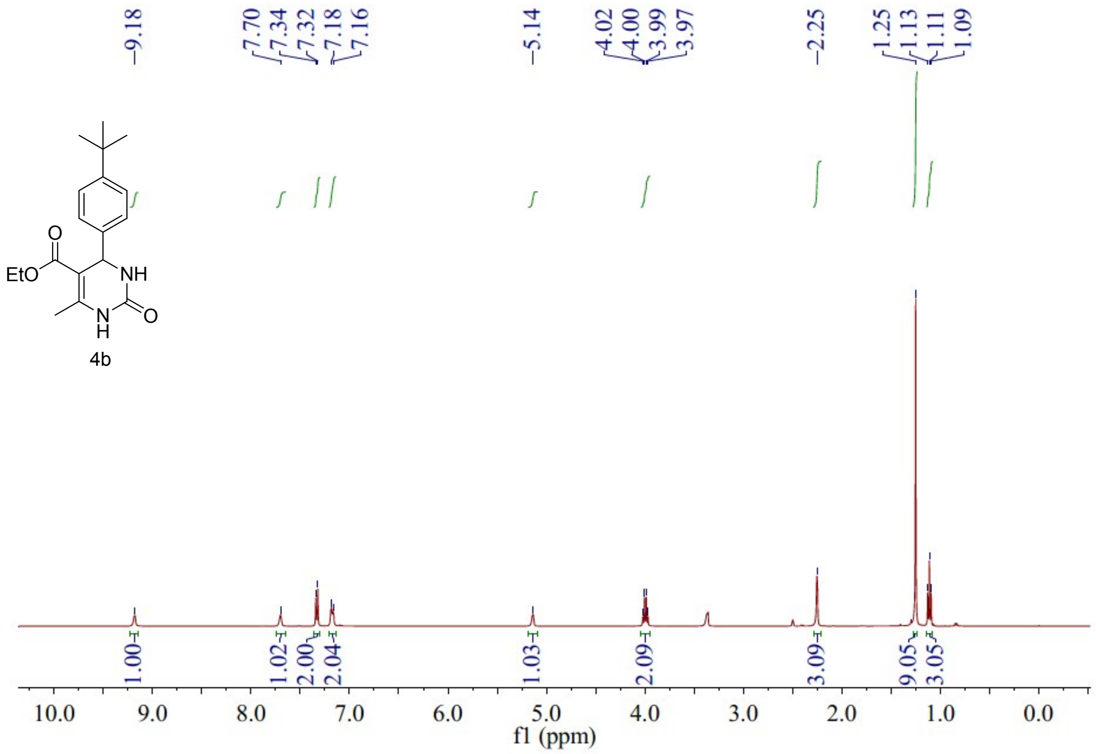

9.18
7.70
7.34
7.32
7.18
7.16
5.14
4.02
4.00
3.99
3.97
2.25
1.25
1.13
1.11
1.09
EtO
O
NH
N
H
O
1.00
1.02
2.00
2.04
1.03
2.09
3.09
9.05
3.05
10.0
9.0
8.0
7.0
6.0
5.0
4.0
3.0
2.0
1.0
0.0
f1 (ppm)


4b

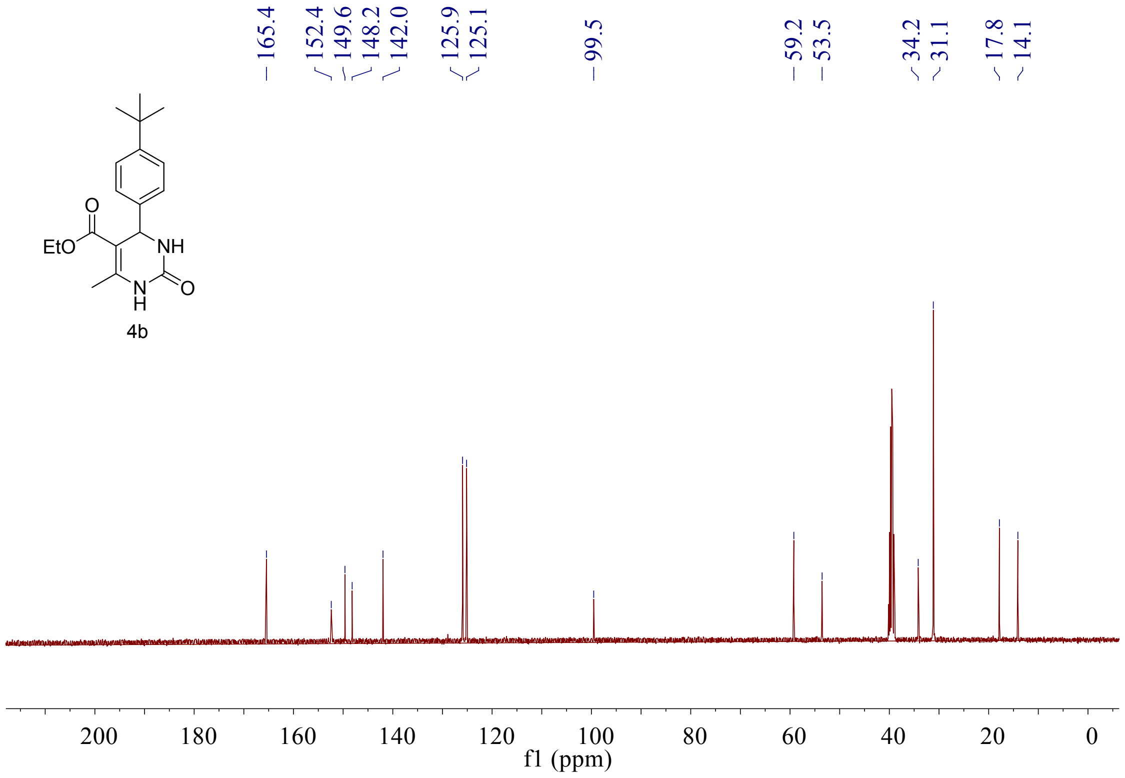

165.4
152.4
149.6
148.2
142.0
125.9
125.1
99.5
59.2
53.5
34.2
31.1
17.8
14.1
EtO
O
NH
N
H
O
200
180
160
140
120
100
80
60
40
20
0
f1 (ppm)


4b

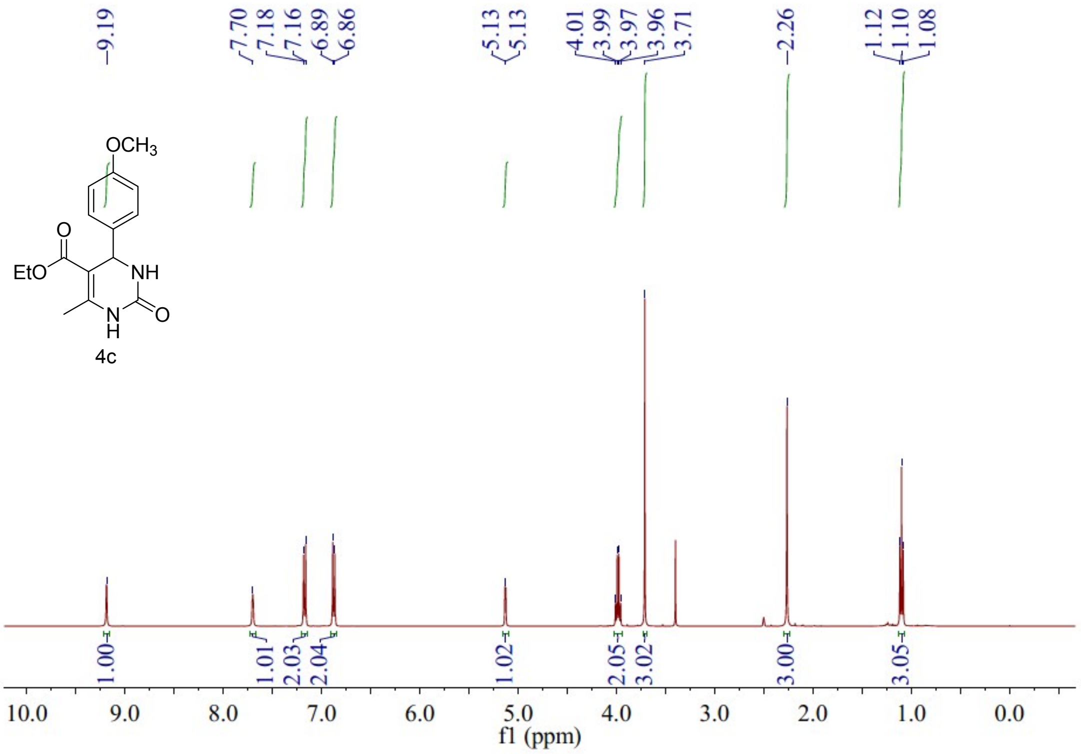

9.19
7.70
7.18
7.16
6.89
6.86
5.13
5.13
4.01
3.99
3.97
3.96
3.71
2.26
1.12
1.10
1.08
OCH3
EtO
O
NH
N
H
O
4c
1.00
1.01
2.03
2.04
1.02
2.05
3.02
3.00
3.05
10.0
9.0
8.0
7.0
6.0
5.0
4.0
3.0
2.0
1.0
0.0
f1 (ppm)


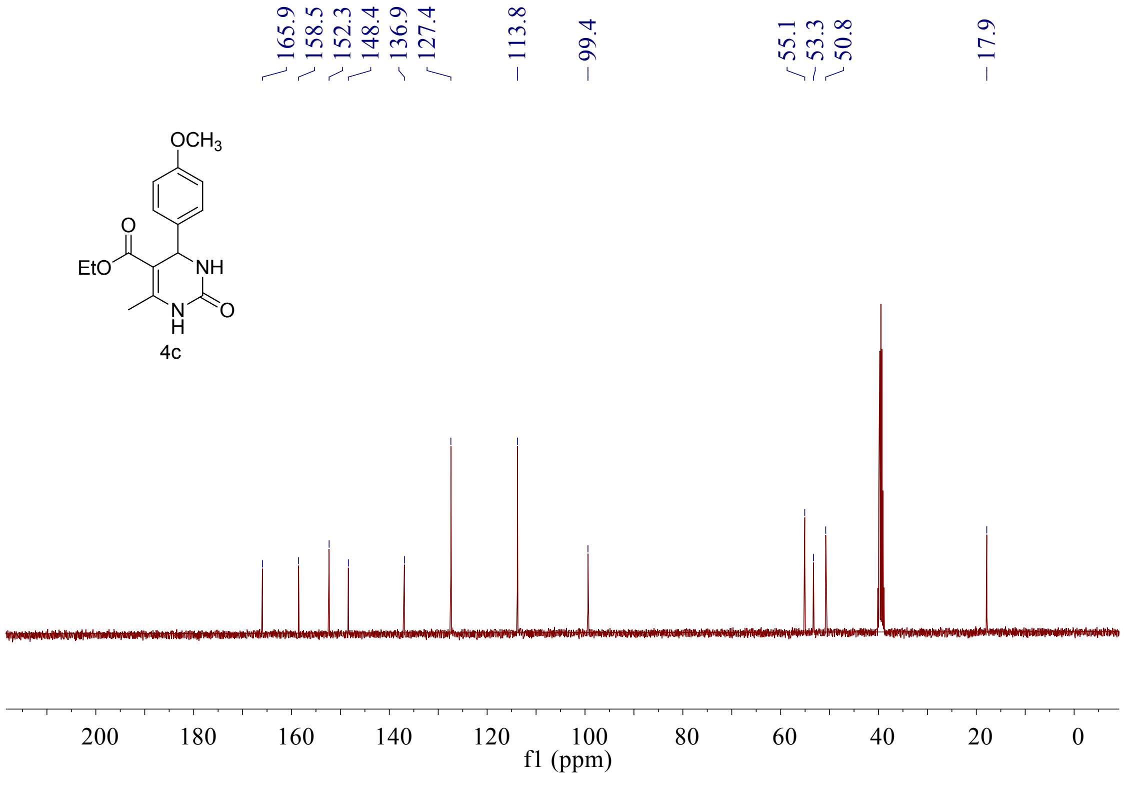

165.9
158.5
152.3
148.4
136.9
127.4
113.8
99.4
55.1
53.3
50.8
17.9
OCH3
EtO
O
NH
N
H
O
4c
200
180
160
140
120
100
80
60
40
20
0
f1 (ppm)

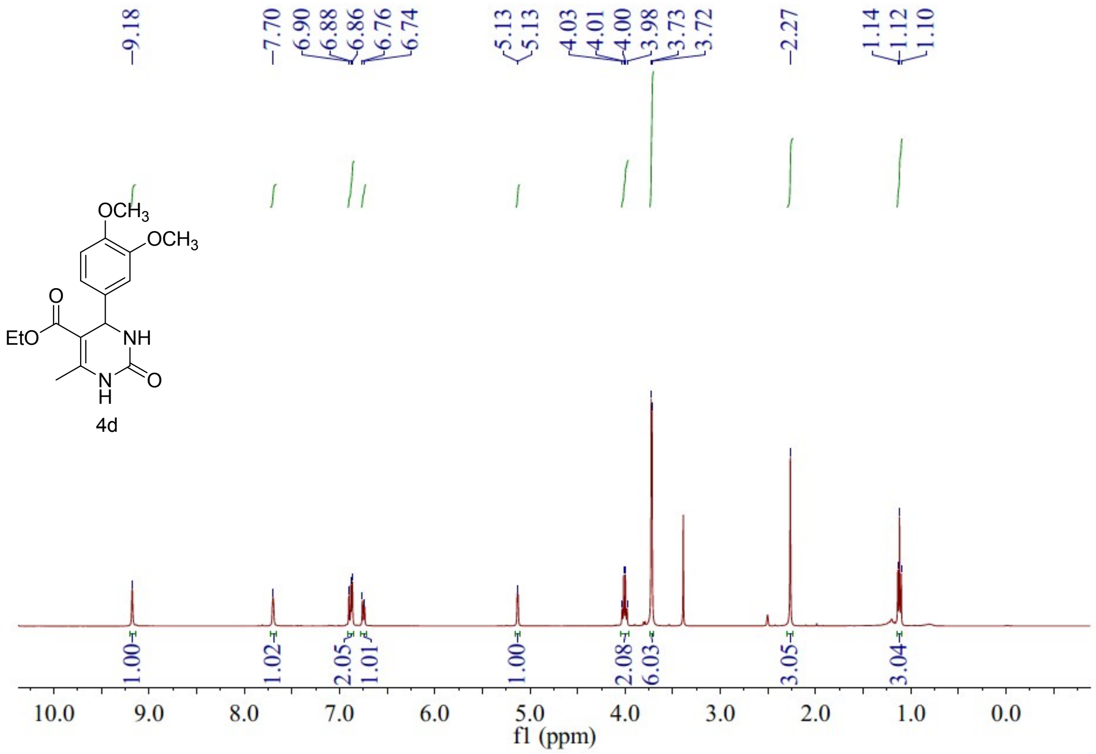
9.18
7.70
6.90
6.88
6.86
6.76
6.74
5.13
5.13
4.03
4.01
4.00
3.98
3.73
3.72
2.27
1.14
1.12
1.10
OCH3
OCH3
O
EtO
NH
N
H
O
4d
1.00
1.02
2.05
1.01
1.00
2.08
6.03
3.05
3.04
10.0
9.0
8.0
7.0
6.0
5.0
4.0
3.0
2.0
1.0
0.0
f1 (ppm)

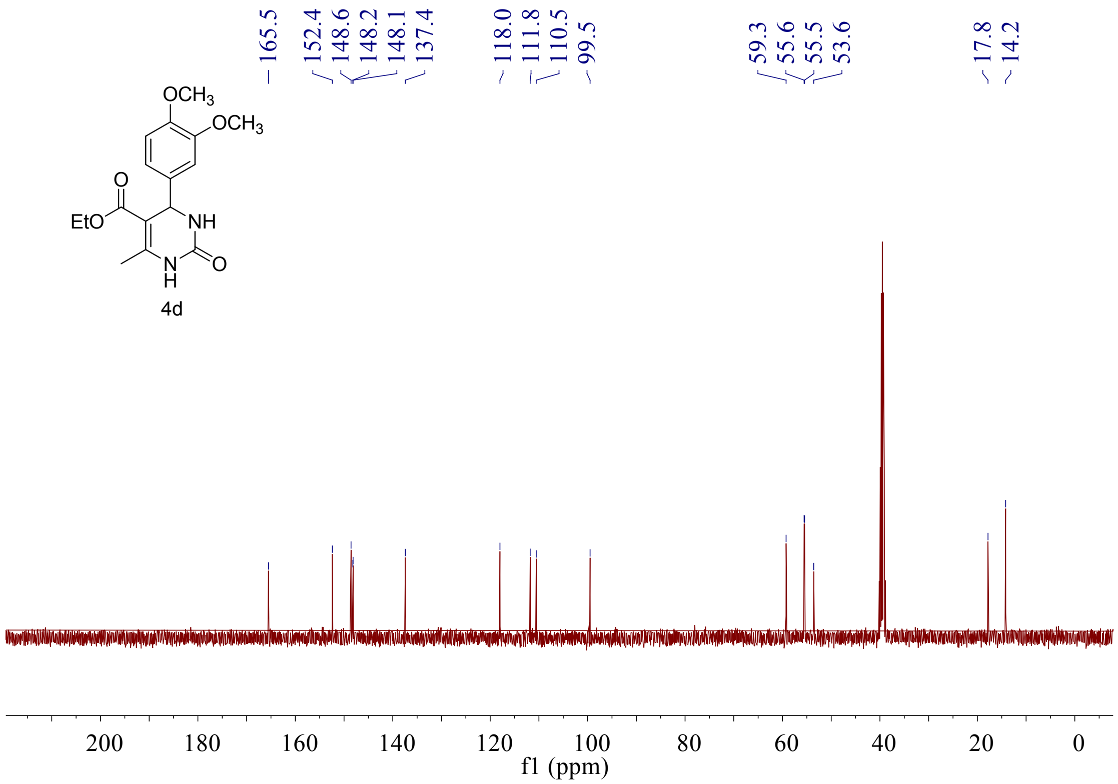
165.5
152.4
148.6
148.2
148.1
137.4
118.0
111.8
110.5
99.5
59.3
55.6
55.5
53.6
17.8
14.2
OCH3
OCH3
O
EtO
NH
N
H
O
4d
200
180
160
140
120
100
80
60
40
20
0
f1 (ppm)

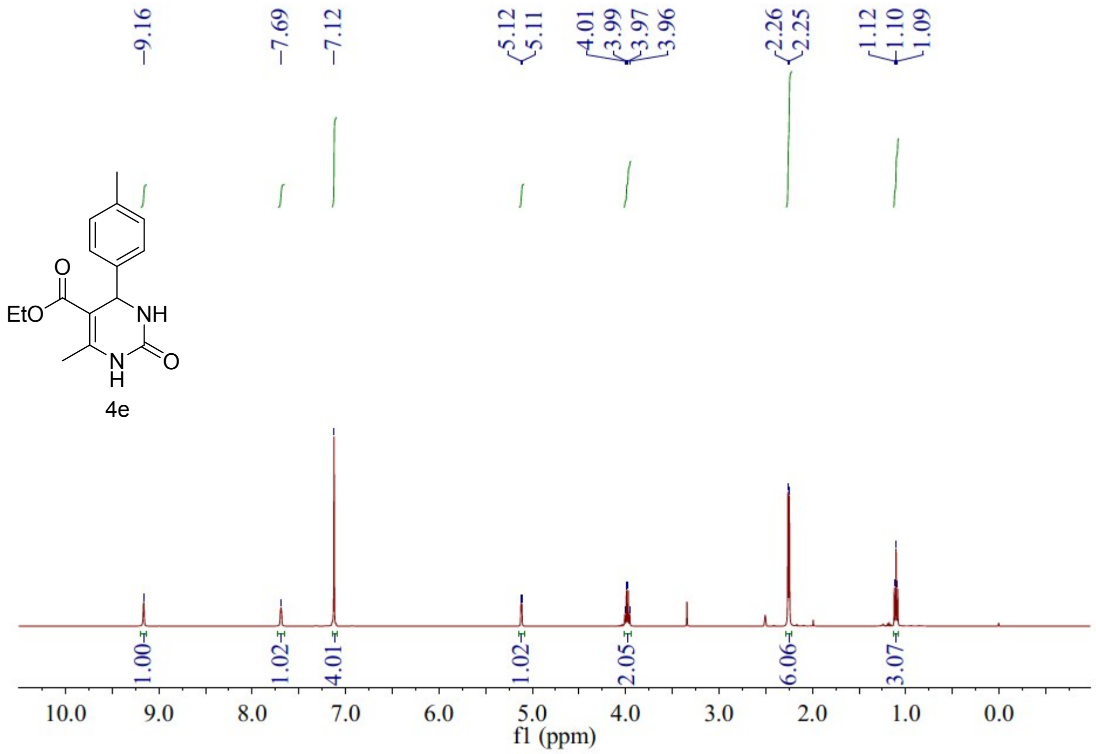

9.16
7.69
7.12
5.12
5.11
4.01
3.99
3.97
3.96
2.26
2.25
1.12
1.10
1.09
EtO
NH
O
N
H
4e
1.00
1.02
4.01
1.02
2.05
6.06
3.07
10.0 9.0 8.0 7.0 6.0 5.0 4.0 3.0 2.0 1.0 0.0
f1 (ppm)


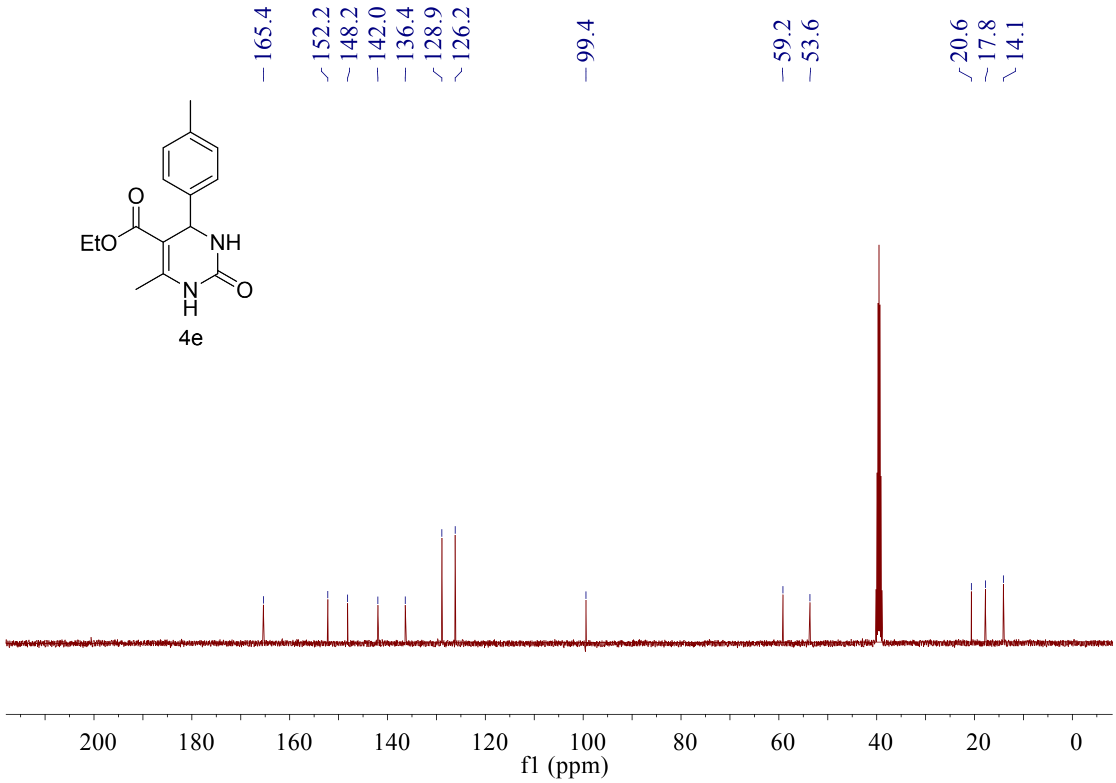

165.4
152.2
148.2
142.0
136.4
128.9
126.2
99.4
59.2
53.6
20.6
17.8
14.1
EtO
NH
O
N
H
4e
200 180 160 140 120 100 80 60 40 20 0
f1 (ppm)

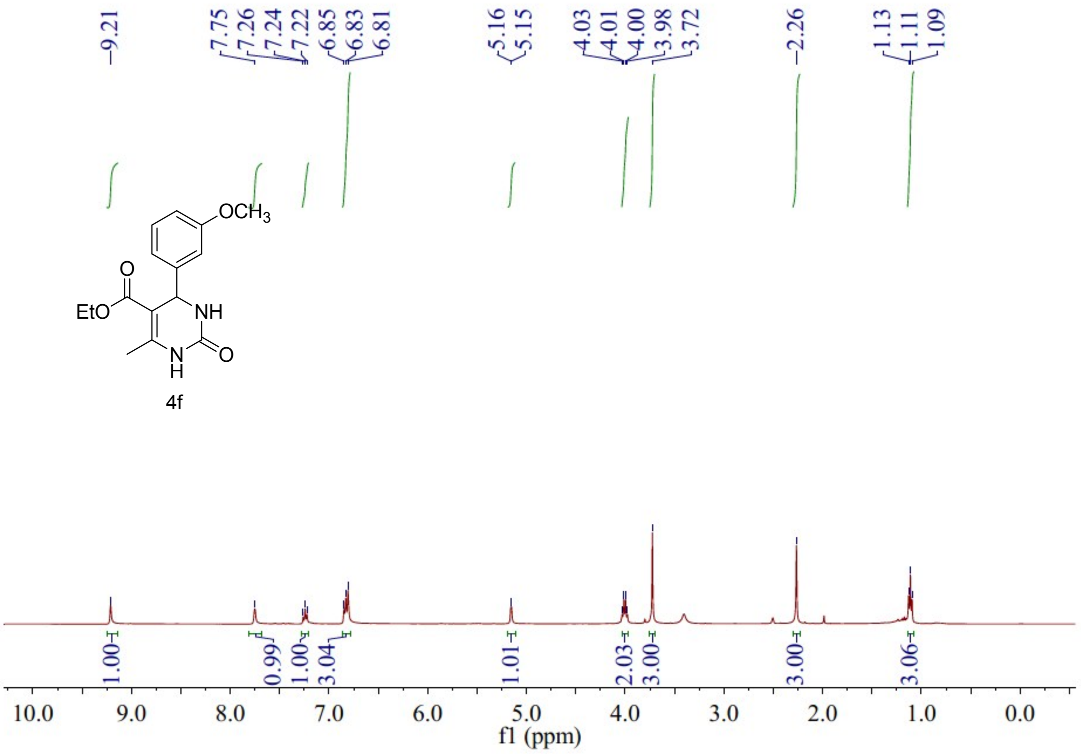
9.21
7.75
7.26
7.24
7.22
6.85
6.83
6.81
5.16
5.15
4.03
4.01
4.00
3.98
3.72
2.26
1.13
1.11
1.09
OCH3
EtO
O
NH
N
H
O
4f
1.00
0.99
1.00
3.04
1.01
2.03
3.00
3.00
3.06
10.0 9.0 8.0 7.0 6.0 5.0 4.0 3.0 2.0 1.0 0.0
f1 (ppm)

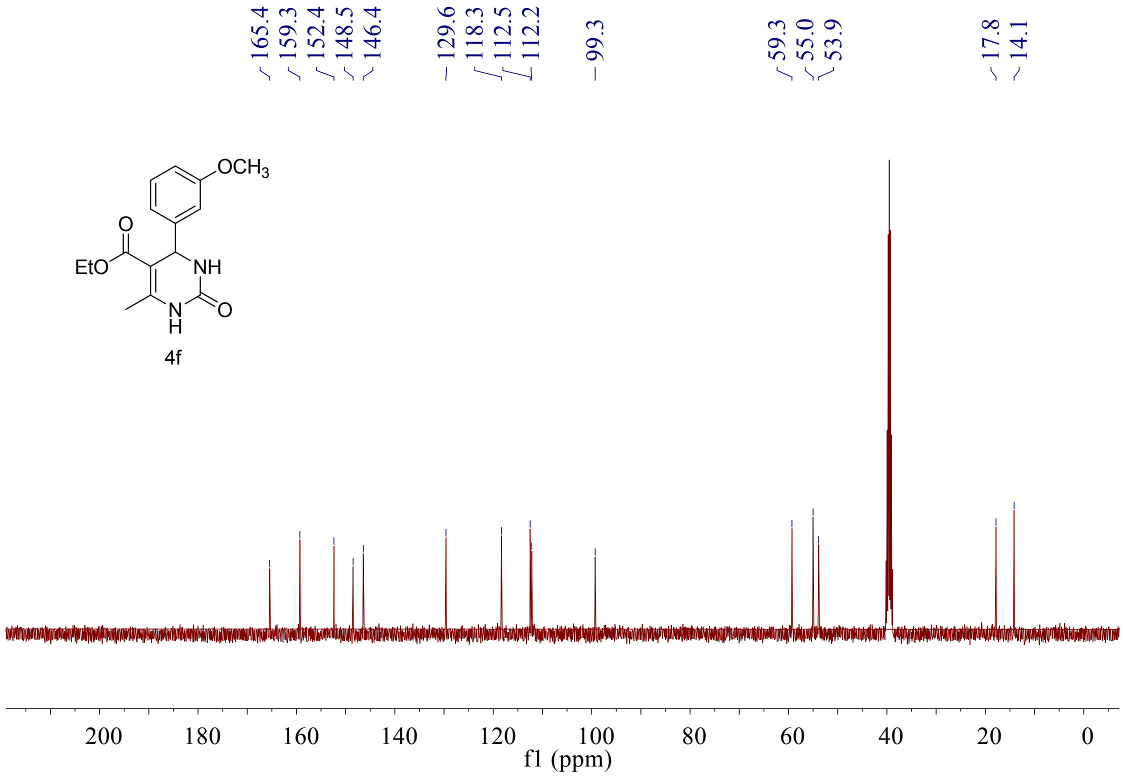
165.4
159.3
152.4
148.5
146.4
129.6
118.3
112.5
112.2
99.3
59.3
55.0
53.9
17.8
14.1
OCH3
EtO
O
NH
N
H
O
4f
200 180 160 140 120 100 80 60 40 20 0
f1 (ppm)

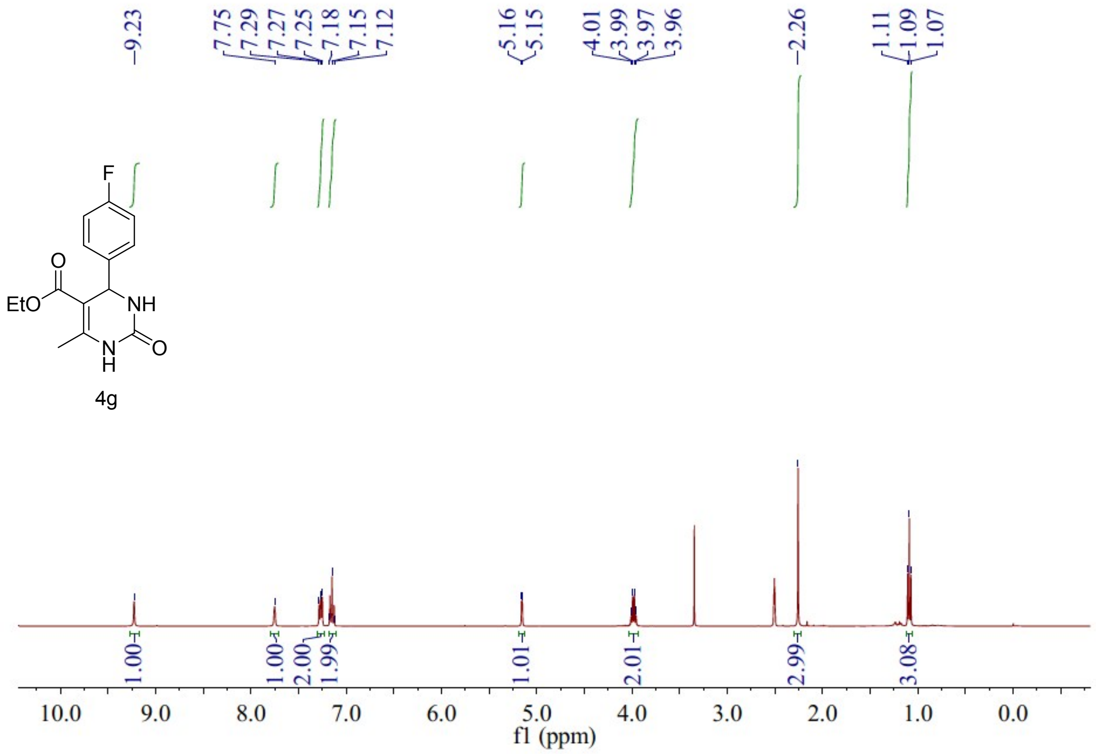
9.23
7.75
7.29
7.27
7.25
7.18
7.15
7.12
5.16
5.15
4.01
3.99
3.97
3.96
2.26
1.11
1.09
1.07
F
O
EtO
NH
N
H
O
4g
1.00
1.00
2.00
1.99
1.01
2.01
2.99
3.08
10.0
9.0
8.0
7.0
6.0
5.0
4.0
3.0
2.0
1.0
0.0
f1 (ppm)

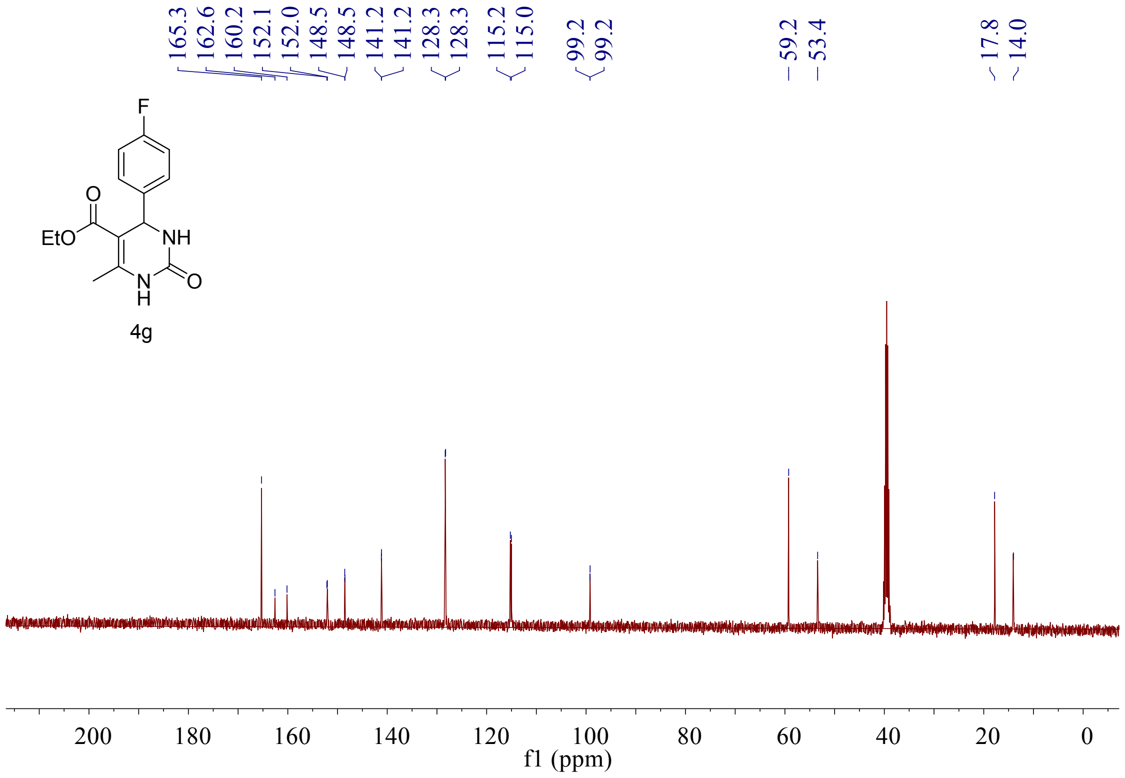
165.3
162.6
160.2
152.1
152.0
148.5
148.5
141.2
141.2
128.3
128.3
115.2
115.0
99.2
99.2
59.2
53.4
17.8
14.0
F
O
EtO
NH
N
H
O
4g
200
180
160
140
120
100
80
60
40
20
0
f1 (ppm)

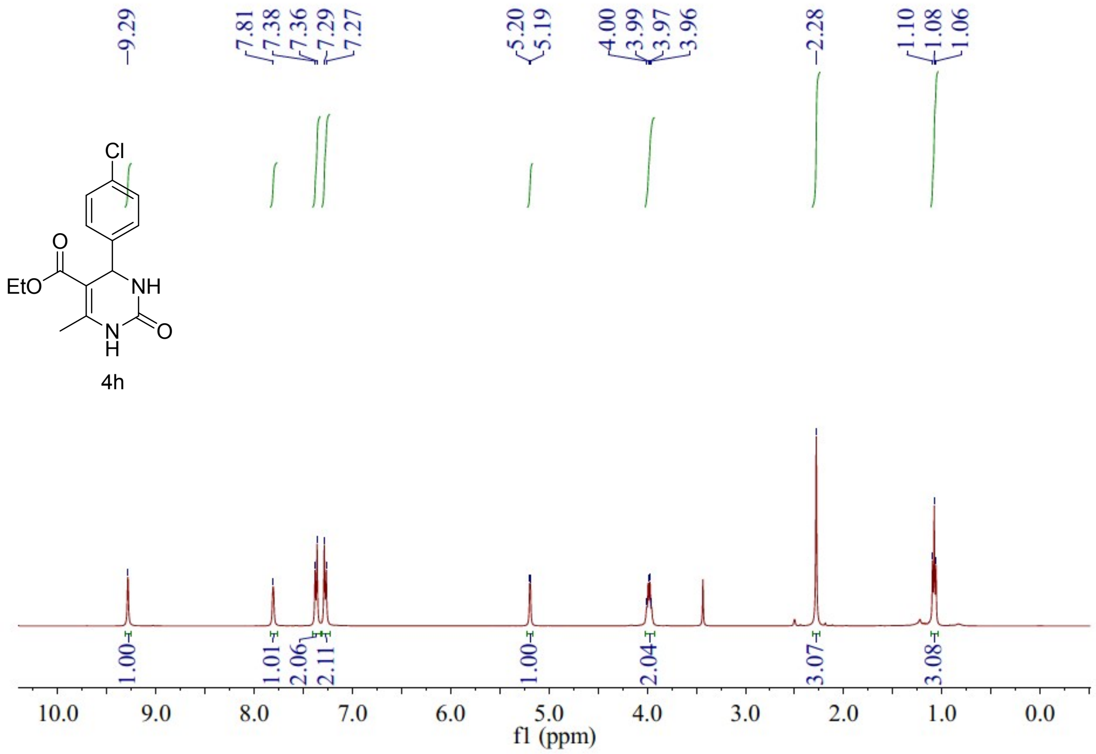
9.29
7.81
7.38
7.36
7.29
7.27
5.20
5.19
4.00
3.99
3.97
3.96
2.28
1.10
1.08
1.06
Cl
O
EtO
NH
N
H
O
4h
1.00
1.01
2.06
2.11
1.00
2.04
3.07
3.08
10.0
9.0
8.0
7.0
6.0
5.0
4.0
3.0
2.0
1.0
0.0
f1 (ppm)

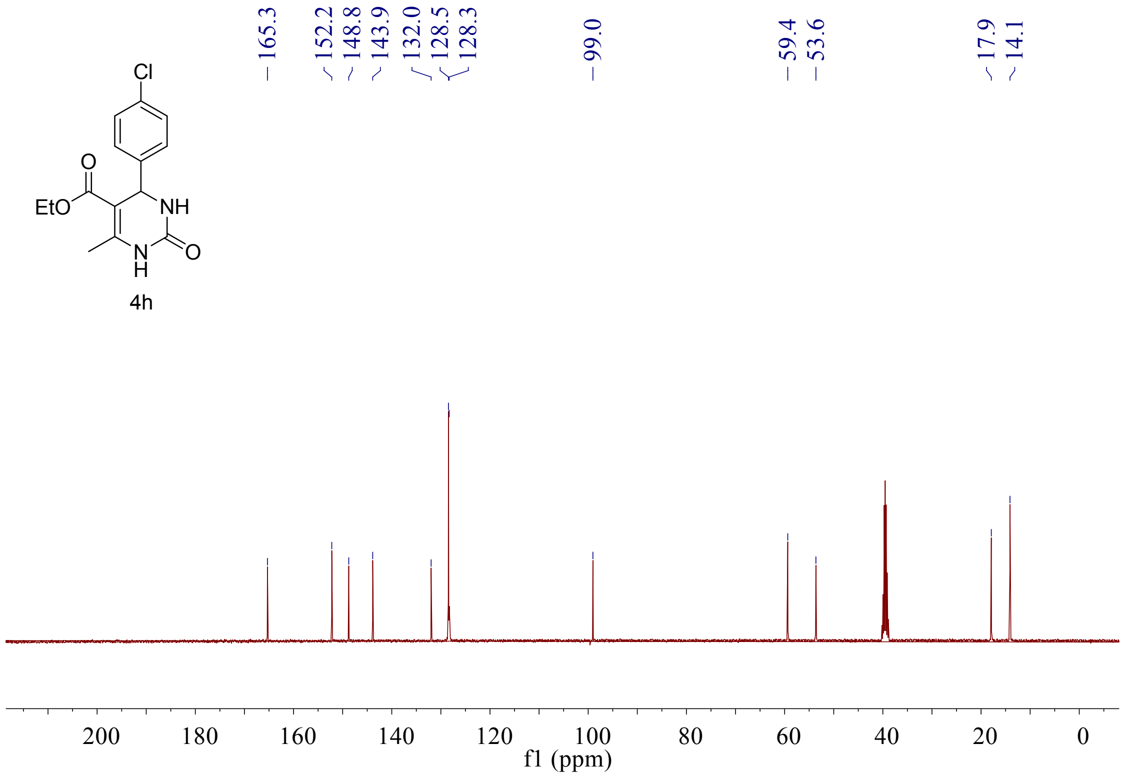
165.3
152.2
148.8
143.9
132.0
128.5
128.3
99.0
59.4
53.6
17.9
14.1
Cl
O
EtO
NH
N
H
O
4h
200
180
160
140
120
100
80
60
40
20
0
f1 (ppm)

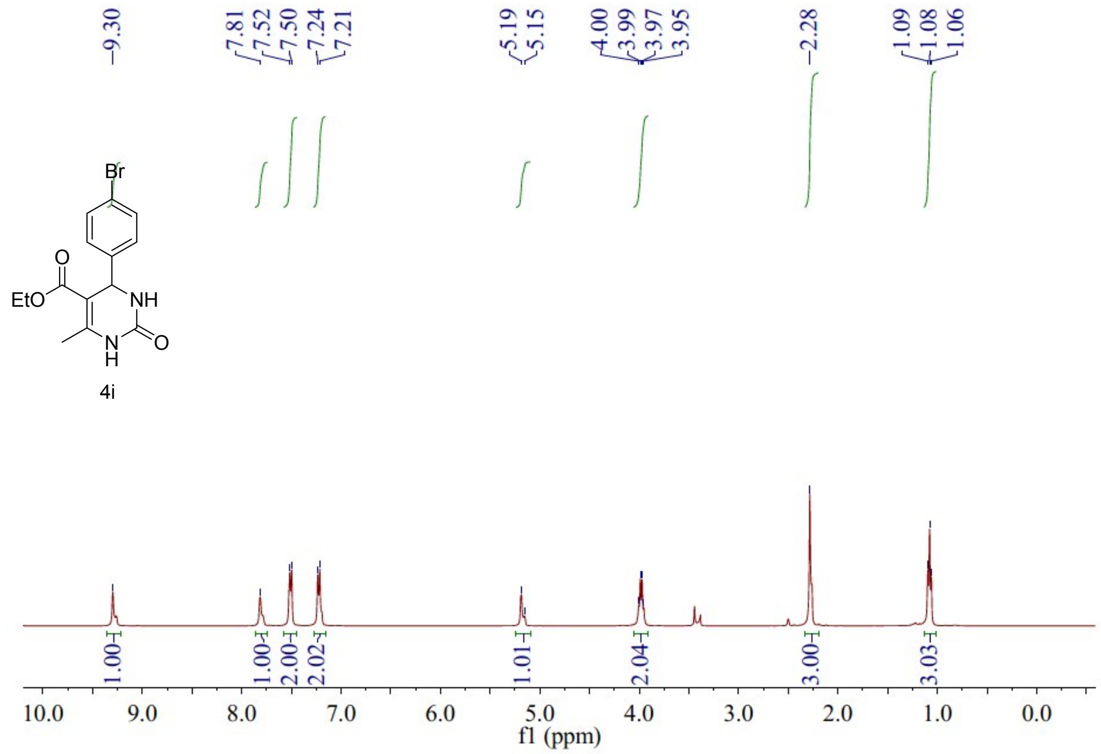

9.30
7.81
7.52
7.50
7.24
7.21
5.19
5.15
4.00
3.99
3.97
3.95
2.28
1.09
1.08
1.06
Br
O
EtO
NH
N
H
O
4i
1.00
1.00
2.00
2.02
1.01
2.04
3.00
3.03
10.0
9.0
8.0
7.0
6.0
5.0
4.0
3.0
2.0
1.0
0.0
f1 (ppm)


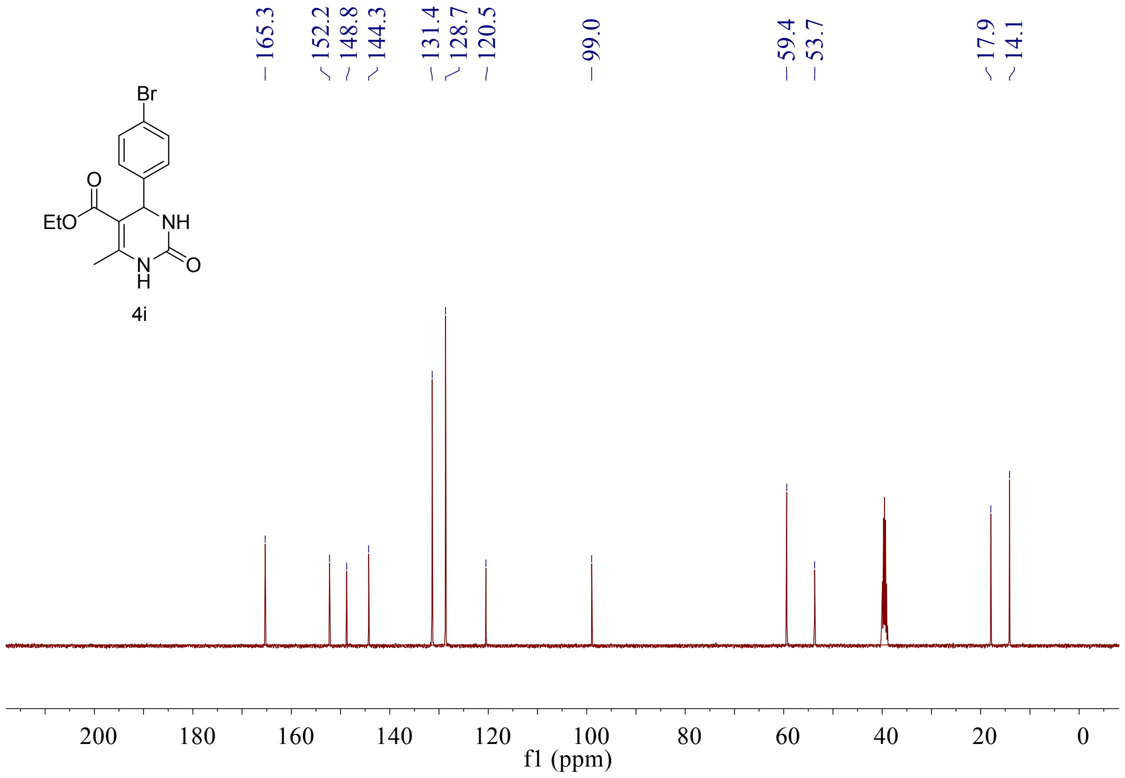

165.3
152.2
148.8
144.3
131.4
128.7
120.5
99.0
59.4
53.7
17.9
14.1
Br
O
EtO
NH
N
H
O
4i
200
180
160
140
120
100
80
60
40
20
0
f1 (ppm)

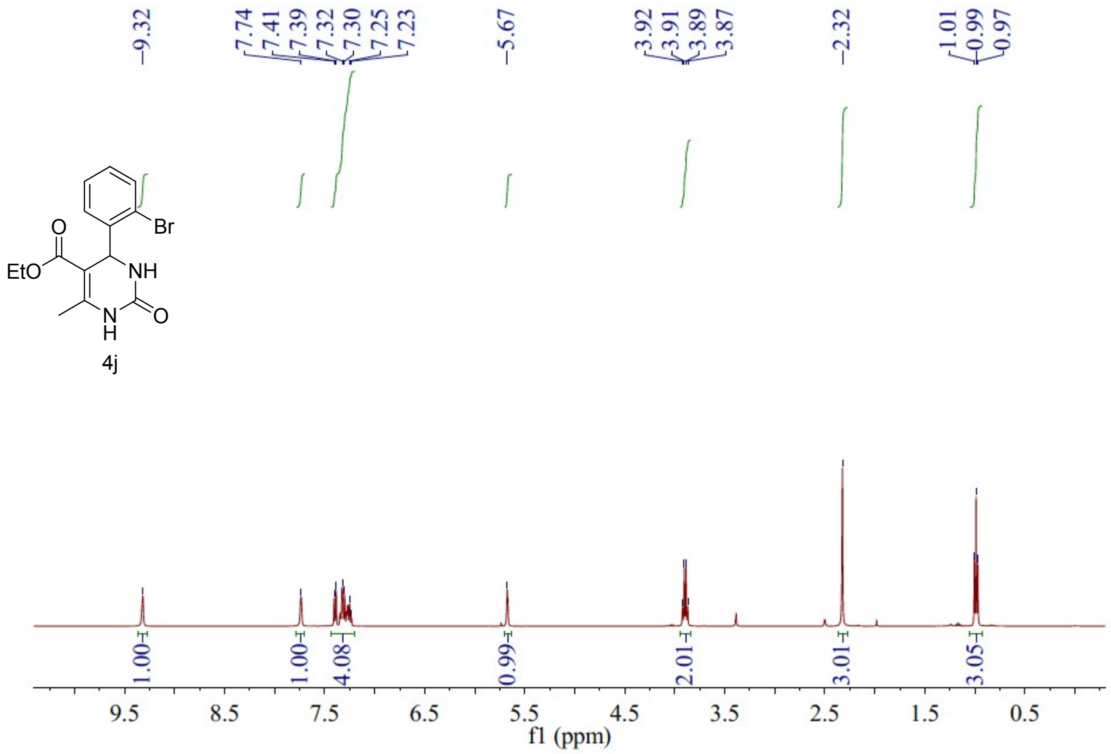
EtO
O
Br
NH
N
H
O
9.32
7.74
7.41
7.39
7.32
7.30
7.25
7.23
5.67
3.92
3.91
3.89
3.87
2.32
1.01
0.99
0.97
1.00
1.00
4.08
0.99
2.01
3.01
3.05
9.5
8.5
7.5
6.5
5.5
4.5
3.5
2.5
1.5
0.5
f1 (ppm)

4j

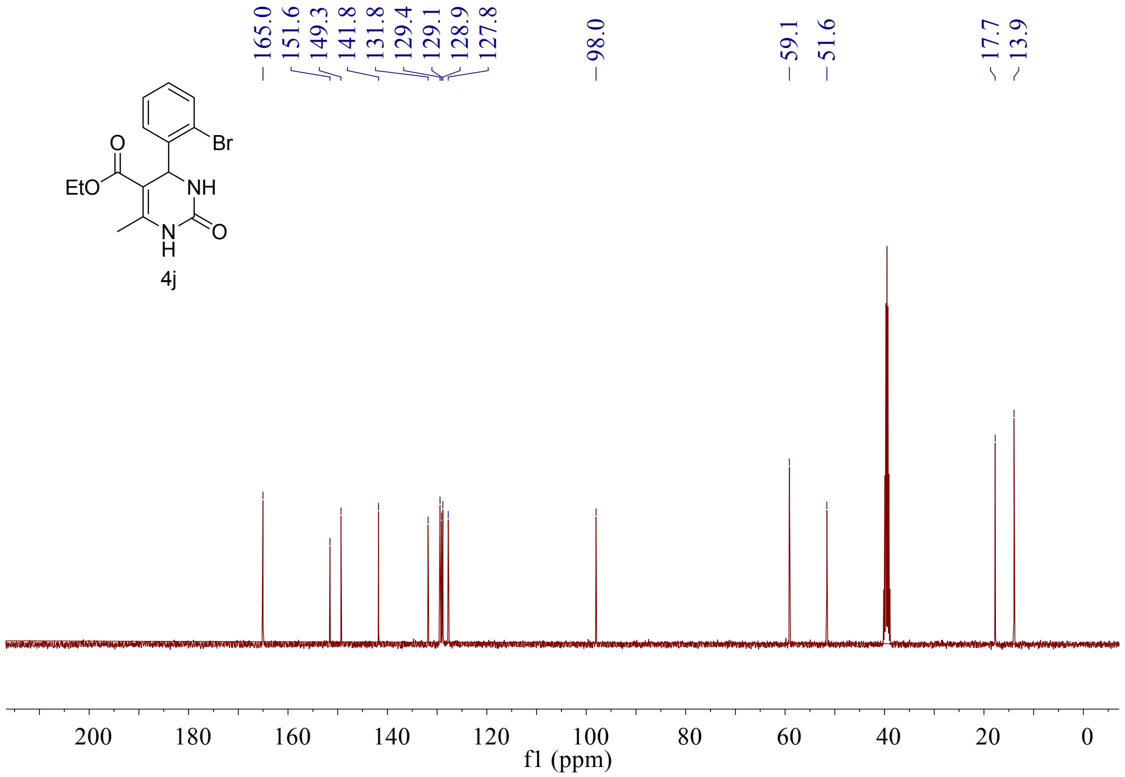
EtO
O
Br
NH
N
H
O
165.0
151.6
149.3
141.8
131.8
129.4
129.1
128.9
127.8
98.0
59.1
51.6
17.7
13.9
200
180
160
140
120
100
80
60
40
20
0
f1 (ppm)

4j

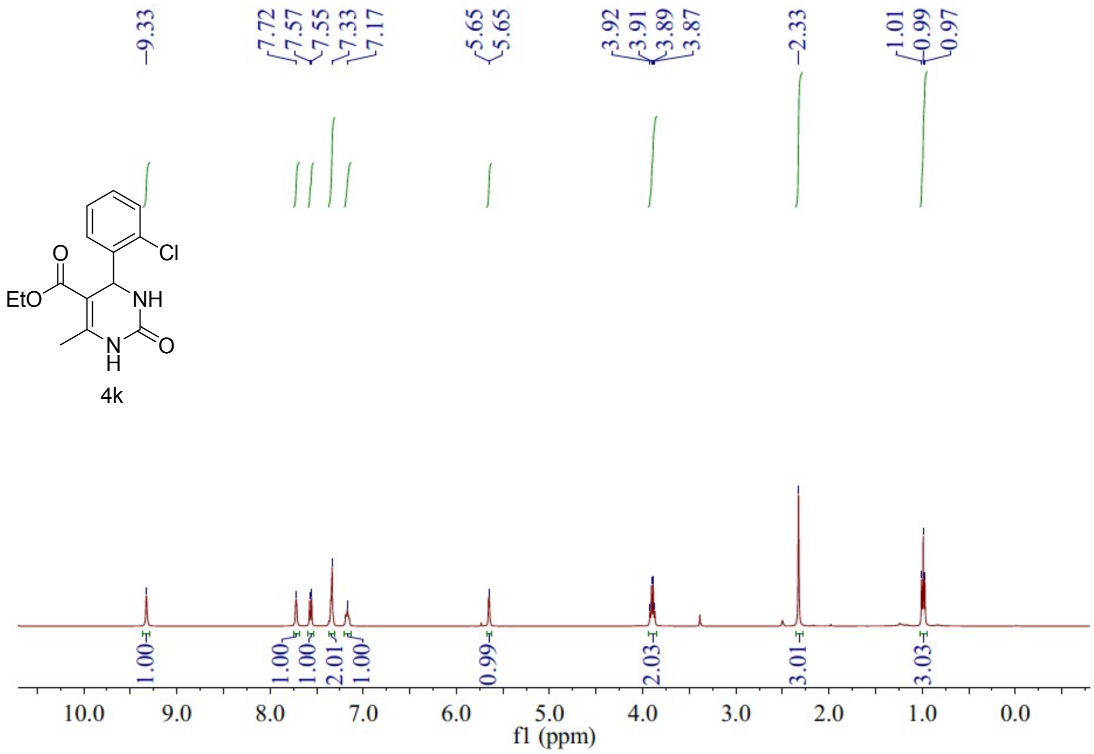
9.33
7.72
7.57
7.55
7.33
7.17
5.65
5.65
3.92
3.91
3.89
3.87
2.33
1.01
0.99
0.97
Cl
O
EtO
NH
N
H
O
4k
1.00
1.00
1.00
2.01
1.00
0.99
2.03
3.01
3.03
10.0
9.0
8.0
7.0
6.0
5.0
4.0
3.0
2.0
1.0
0.0
f1 (ppm)

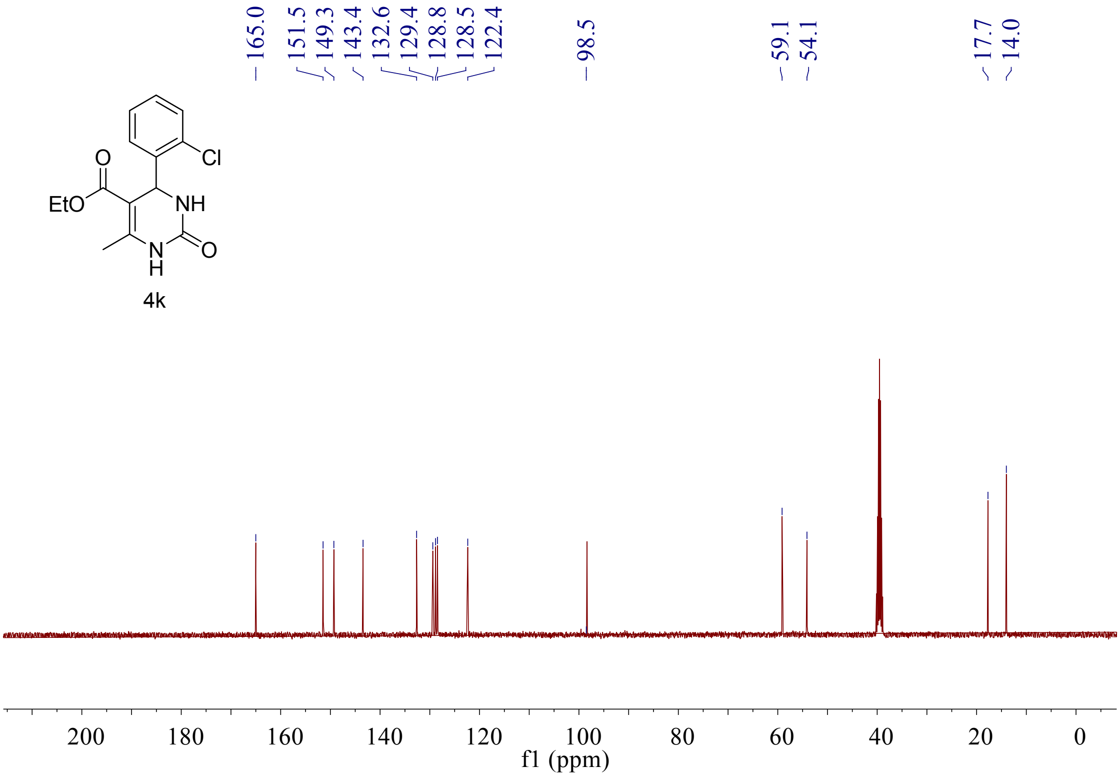
165.0
151.5
149.3
143.4
132.6
129.4
128.8
128.5
122.4
98.5
59.1
54.1
17.7
14.0
Cl
O
EtO
NH
N
H
O
4k
200
180
160
140
120
100
80
60
40
20
0
f1 (ppm)

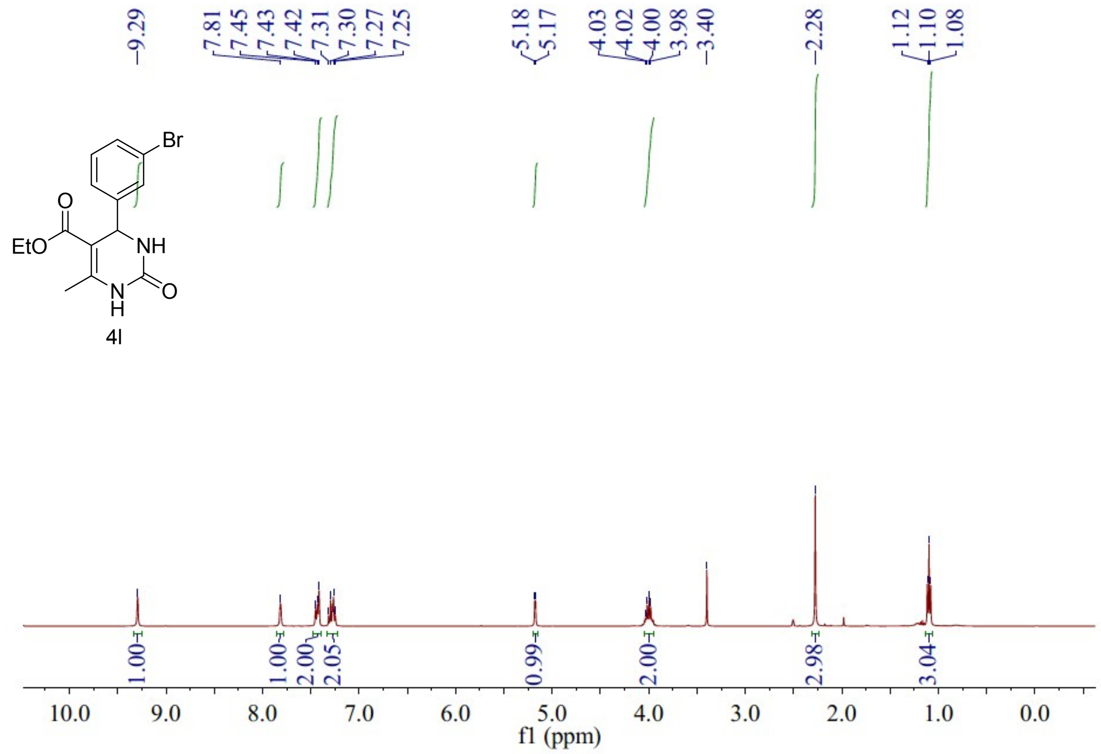
9.29
7.81
7.45
7.43
7.42
7.31
7.30
7.27
7.25
5.18
5.17
4.03
4.02
4.00
3.98
3.40
2.28
1.12
1.10
1.08
Br
O
EtO
NH
N
H
O
1.00
1.00
2.00
2.05
0.99
2.00
2.98
3.04
10.0
9.0
8.0
7.0
6.0
5.0
4.0
3.0
2.0
1.0
0.0
f1 (ppm)

4l

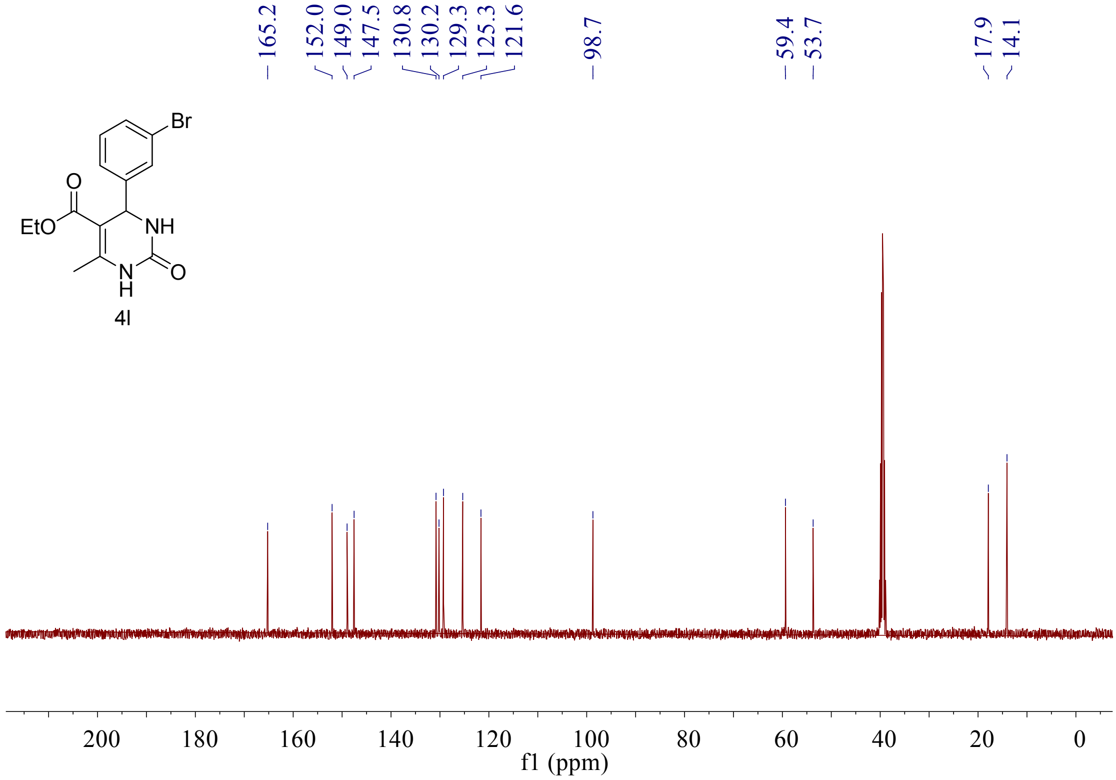
165.2
152.0
149.0
147.5
130.8
130.2
129.3
125.3
121.6
98.7
59.4
53.7
17.9
14.1
Br
O
EtO
NH
N
H
O
200
180
160
140
120
100
80
60
40
20
0
f1 (ppm)

4l

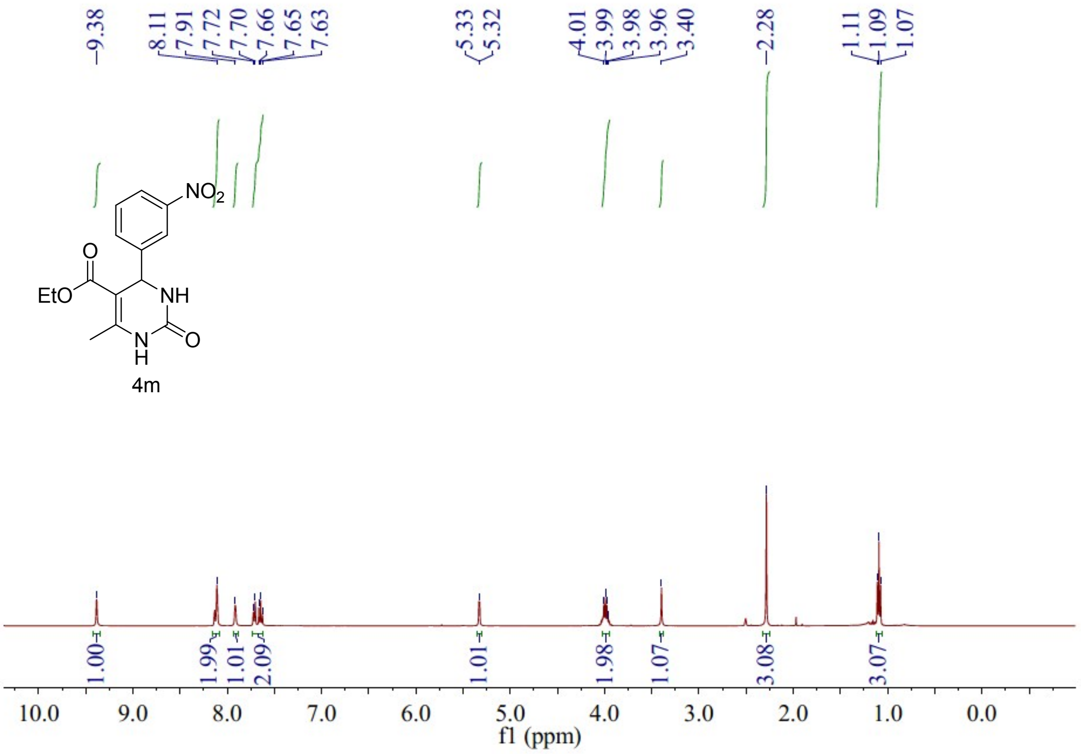
9.38
8.11
7.91
7.72
7.70
7.66
7.65
7.63
5.33
5.32
4.01
3.99
3.98
3.96
3.40
2.28
1.11
1.09
1.07
NO2
O
EtO
NH
N
H
O
4m
1.00
1.99
1.01
2.09
1.01
1.98
1.07
3.08
3.07
10.0
9.0
8.0
7.0
6.0
5.0
4.0
3.0
2.0
1.0
0.0
f1 (ppm)

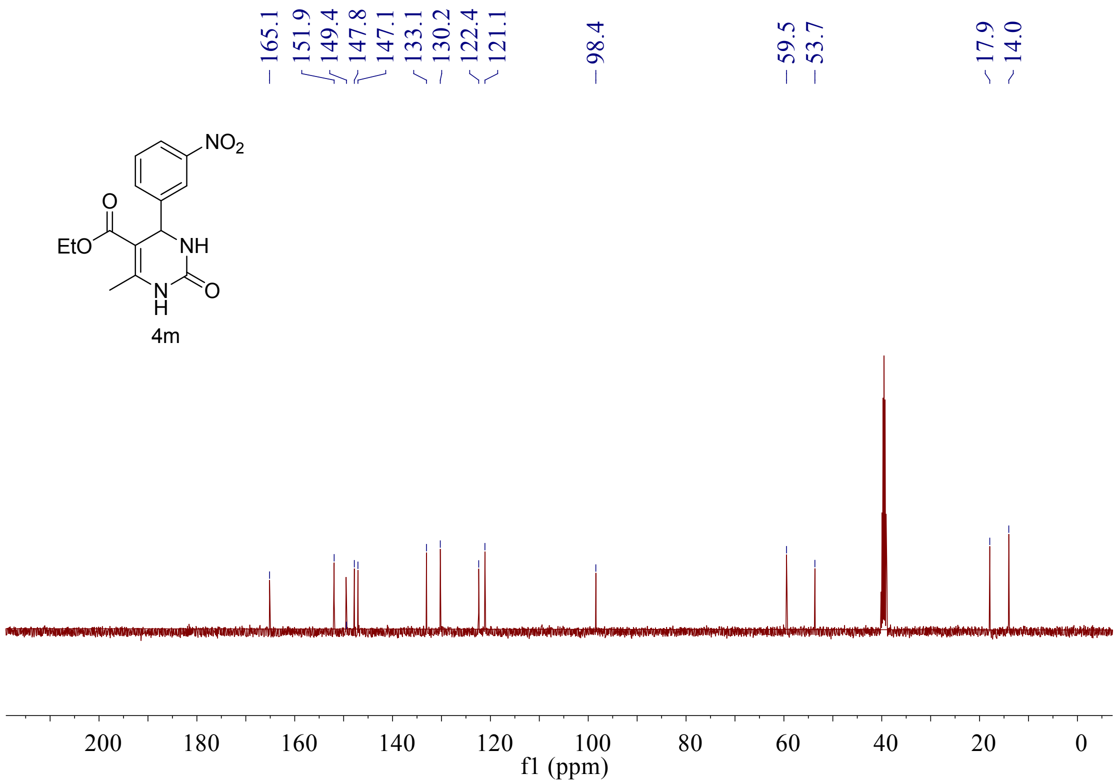
165.1
151.9
149.4
147.8
147.1
133.1
130.2
122.4
121.1
98.4
59.5
53.7
17.9
14.0
NO2
O
EtO
NH
N
H
O
4m
200
180
160
140
120
100
80
60
40
20
0
f1 (ppm)

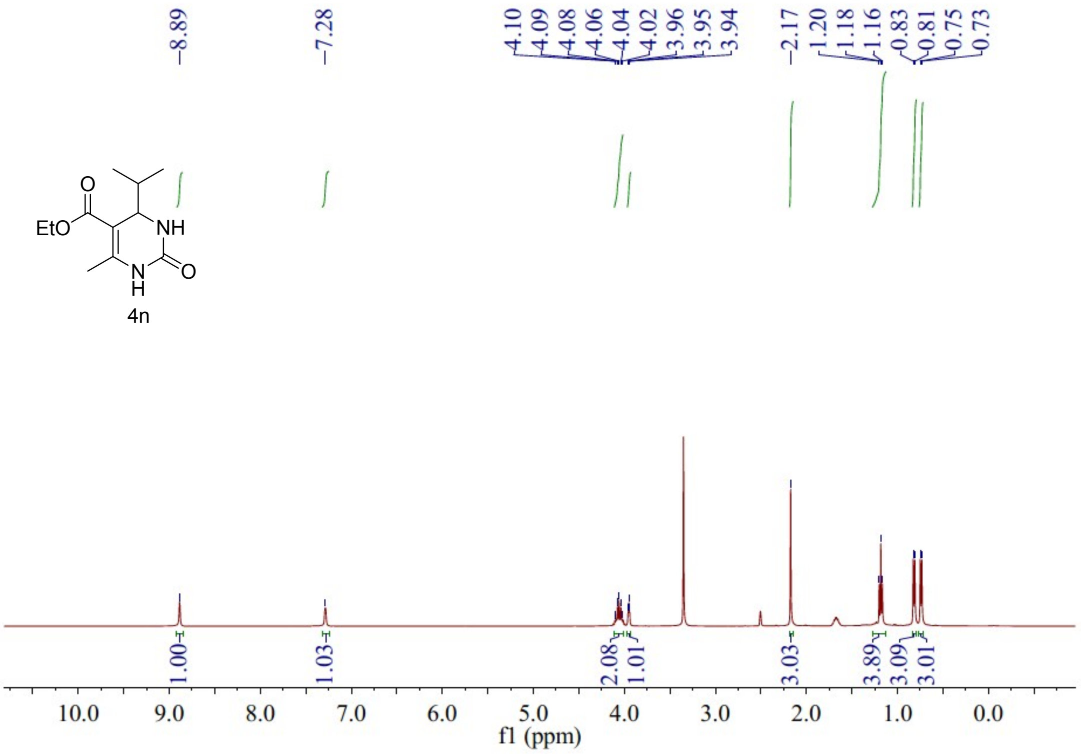

8.89
7.28
4.10
4.09
4.08
4.06
4.04
4.02
3.96
3.95
3.94
2.17
1.20
1.18
1.16
0.83
0.81
0.75
0.73
EtO
O
NH
N
H
O
4n
1.00
1.03
2.08
1.01
3.03
3.89
3.09
3.01
10.0
9.0
8.0
7.0
6.0
5.0
4.0
3.0
2.0
1.0
0.0
f1 (ppm)


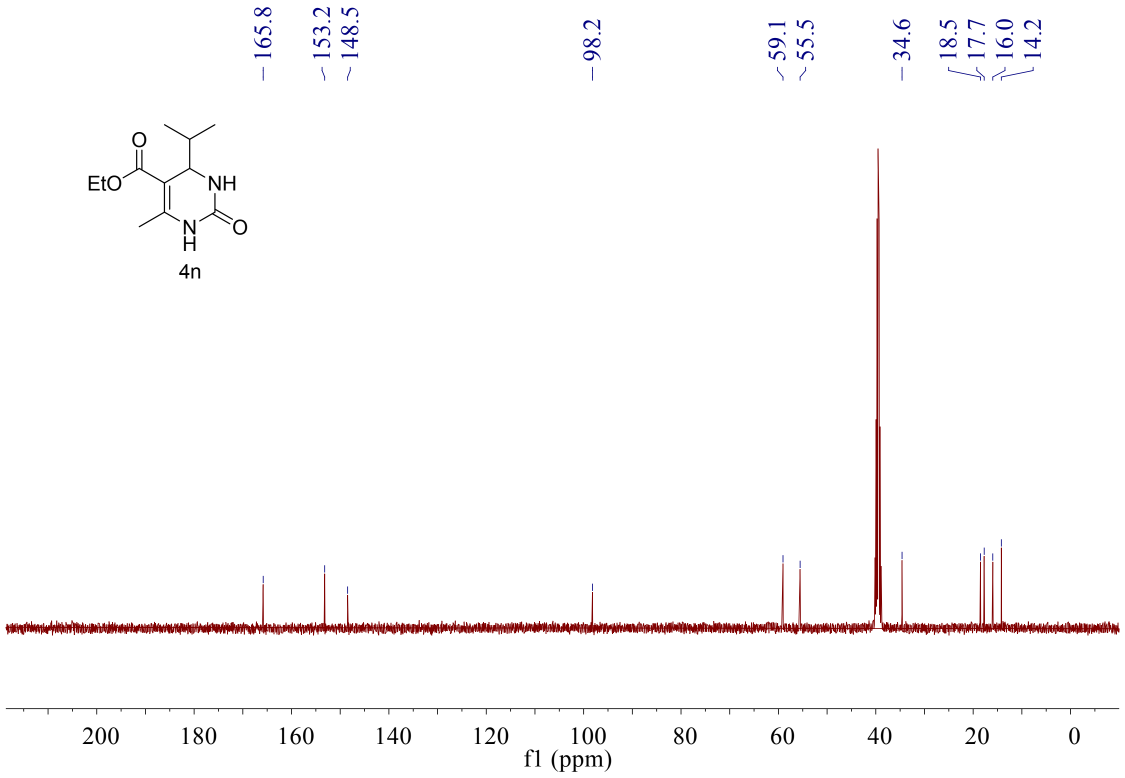

165.8
153.2
148.5
98.2
59.1
55.5
34.6
18.5
17.7
16.0
14.2
EtO
O
NH
N
H
O
4n
200
180
160
140
120
100
80
60
40
20
0
f1 (ppm)

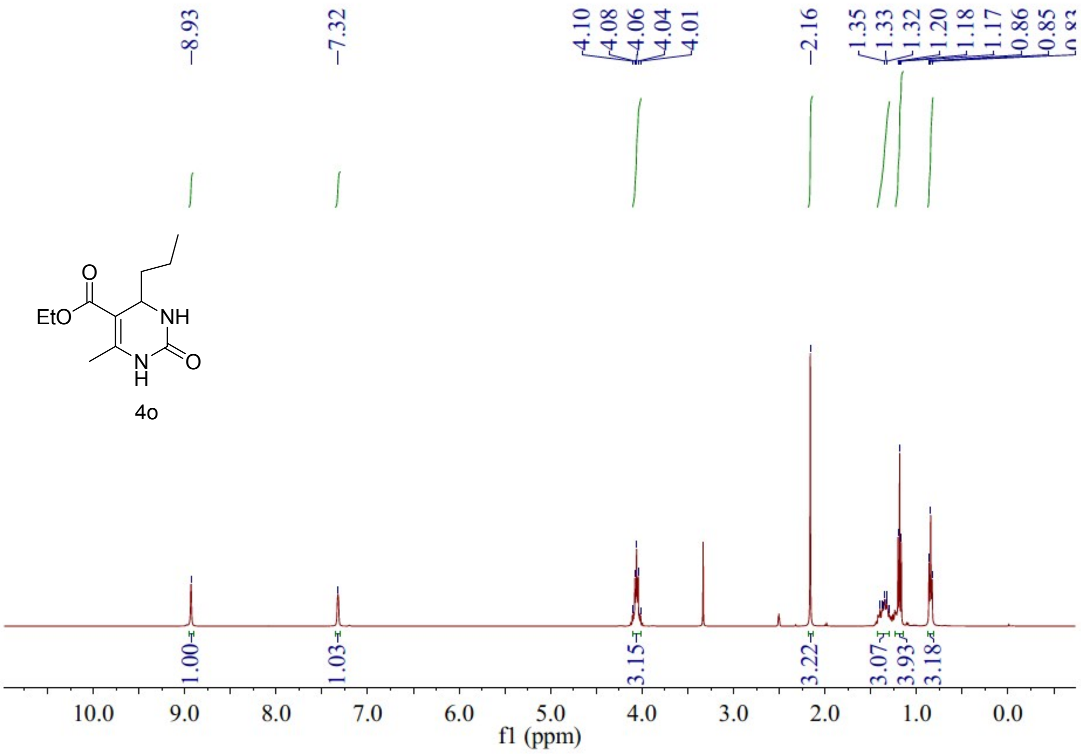

8.93
7.32
4.10
4.08
4.06
4.04
4.01
2.16
1.35
1.33
1.32
1.20
1.18
1.17
0.86
0.85
EtO
O
NH
N
H
O
4o
1.00
1.03
3.15
3.22
3.07
3.93
3.18
10.0
9.0
8.0
7.0
6.0
5.0
4.0
3.0
2.0
1.0
0.0
f1 (ppm)


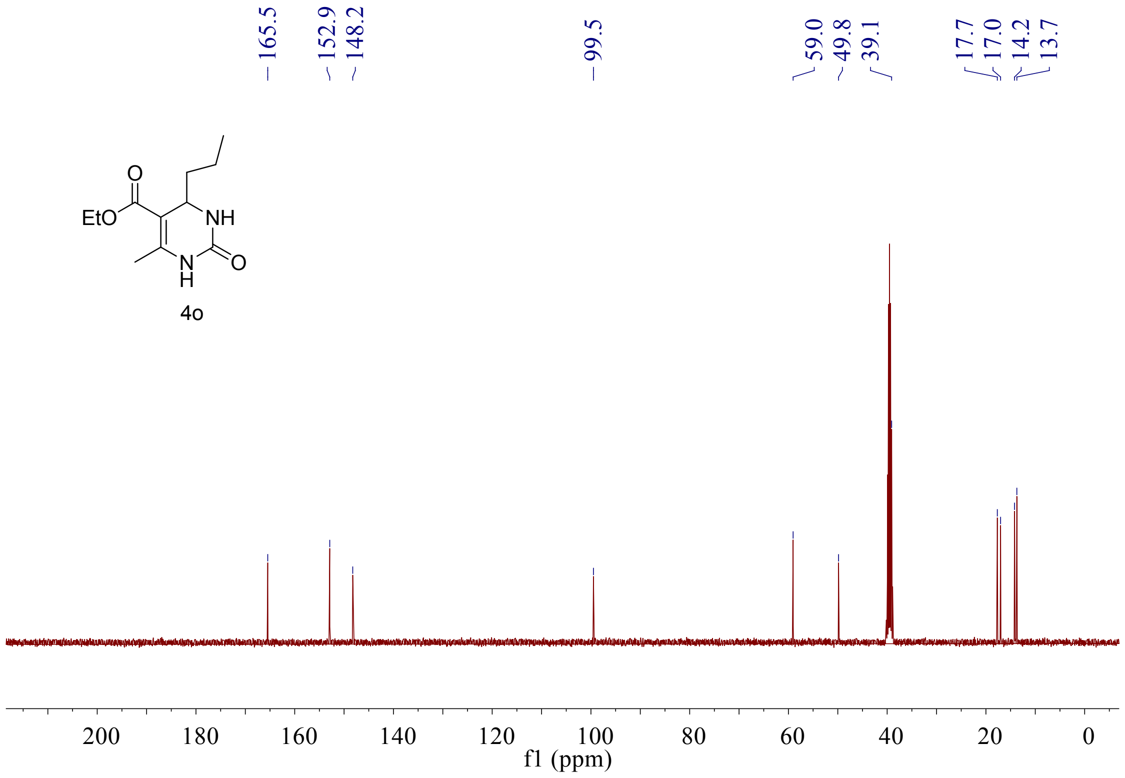

165.5
152.9
148.2
99.5
59.0
49.8
39.1
17.7
17.0
14.2
13.7
EtO
O
NH
N
H
O
4o
200
180
160
140
120
100
80
60
40
20
0
f1 (ppm)

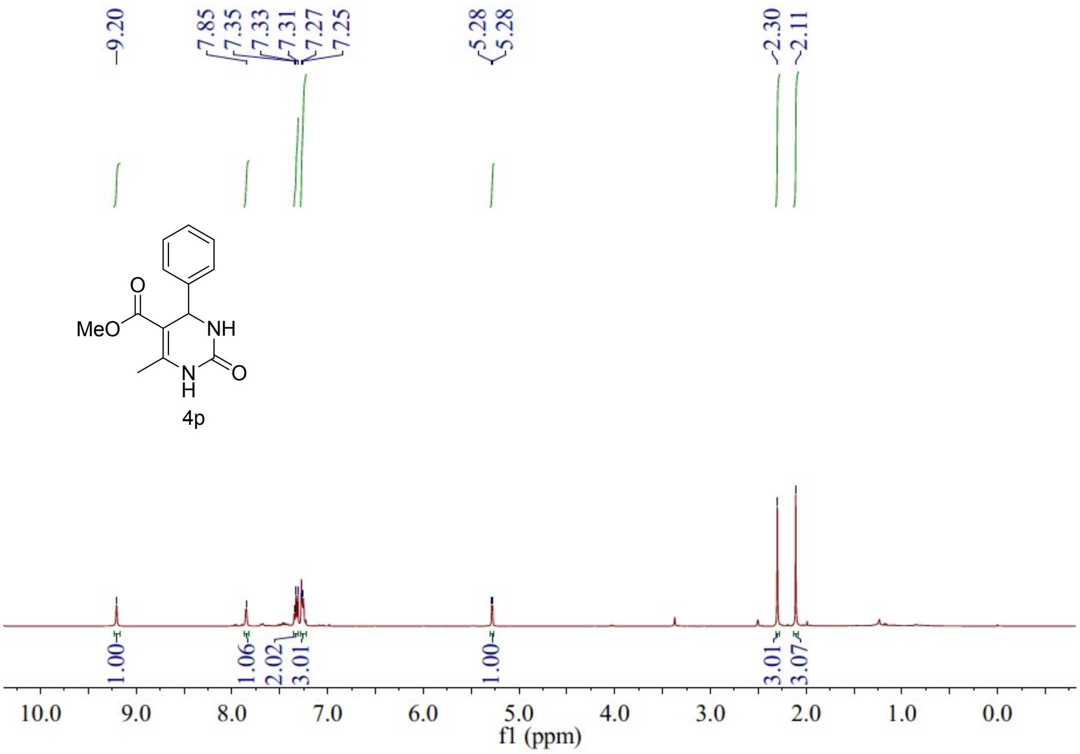
9.20
7.85
7.35
7.33
7.31
7.27
7.25
5.28
5.28
2.30
2.11
MeO
O
NH
N
H
O
4p
1.00
1.06
2.02
3.01
1.00
3.01
3.07
10.0 9.0 8.0 7.0 6.0 5.0 4.0 3.0 2.0 1.0 0.0
f1 (ppm)

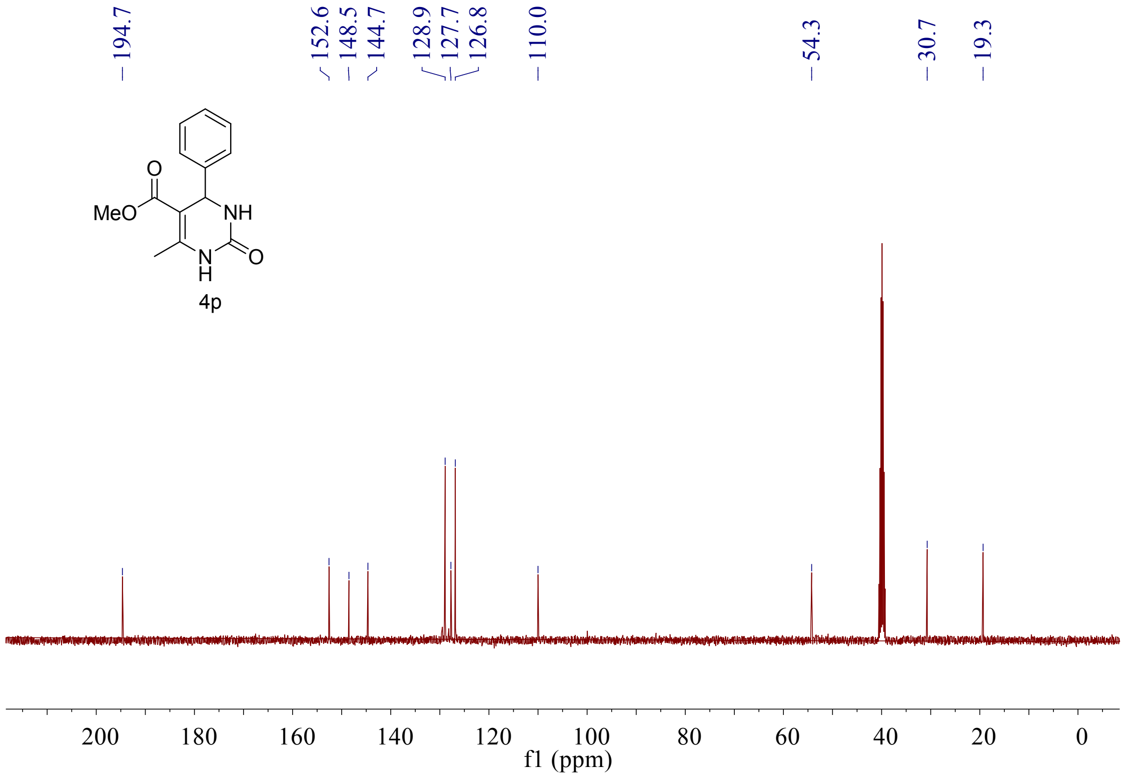
194.7
152.6
148.5
144.7
128.9
127.7
126.8
110.0
54.3
30.7
19.3
MeO
O
NH
N
H
O
4p
200 180 160 140 120 100 80 60 40 20 0
f1 (ppm)

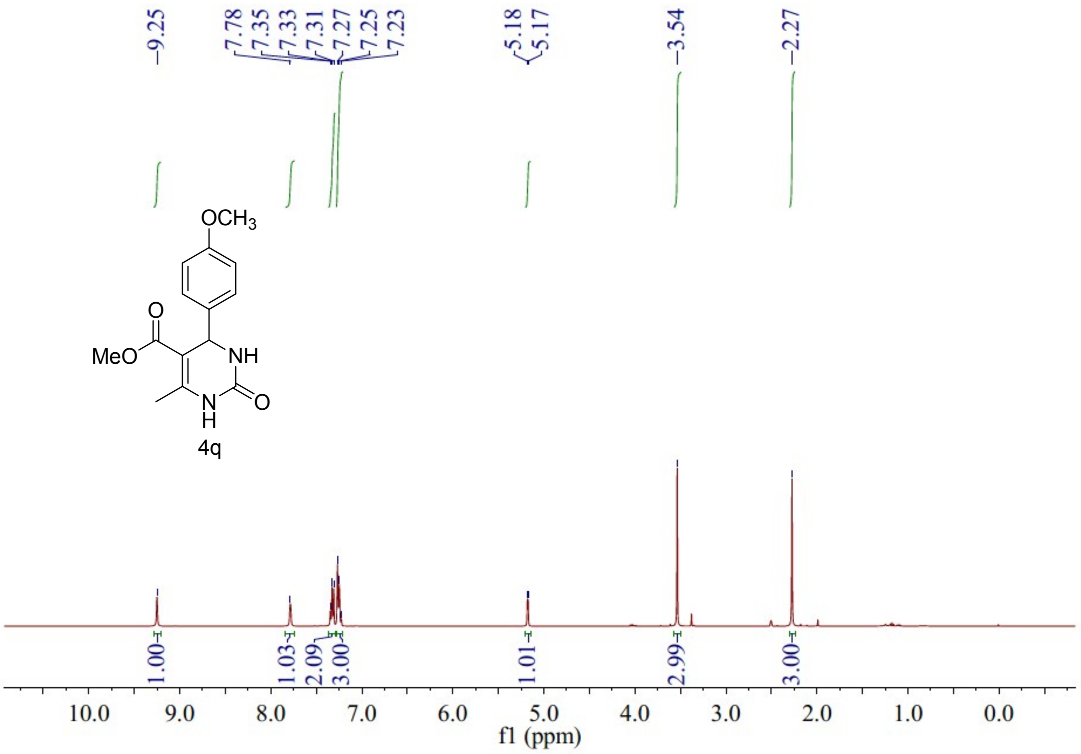
9.25
7.78
7.35
7.33
7.31
7.27
7.25
7.23
5.18
5.17
3.54
2.27
OCH3
O
MeO
NH
N
H
O
4q
1.00
1.03
2.09
3.00
1.01
2.99
3.00
10.0
9.0
8.0
7.0
6.0
5.0
4.0
3.0
2.0
1.0
0.0
f1 (ppm)

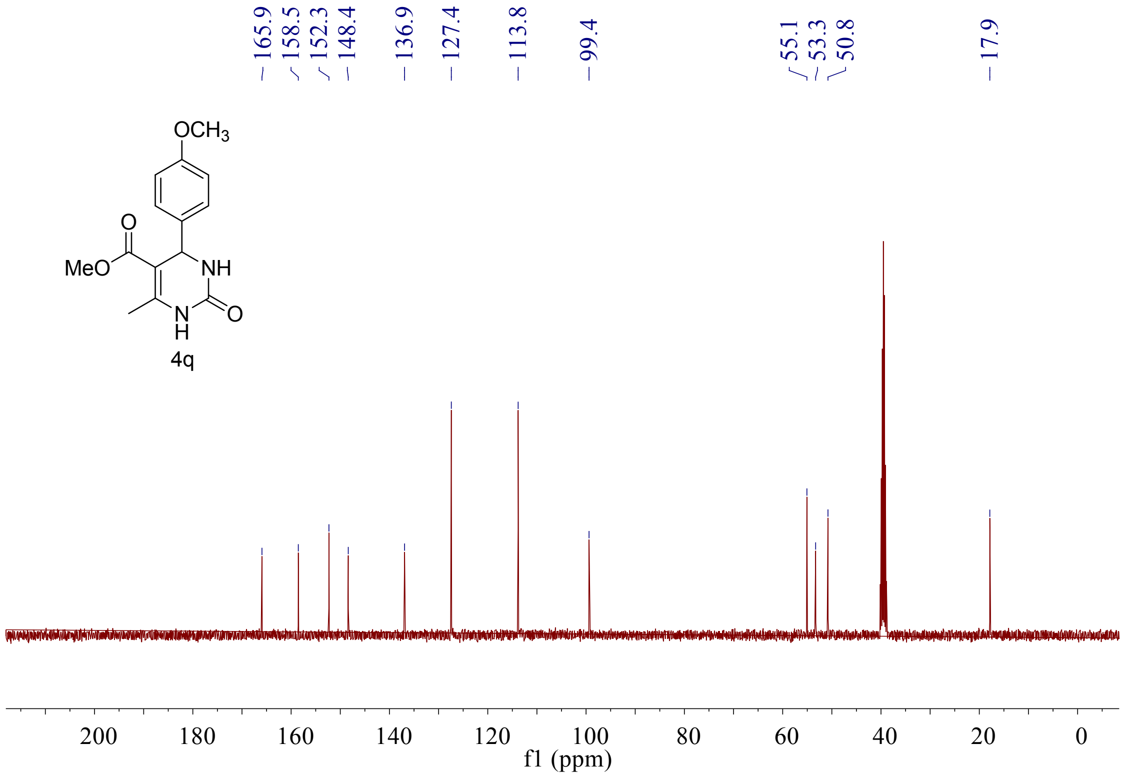
165.9
158.5
152.3
148.4
136.9
127.4
113.8
99.4
55.1
53.3
50.8
17.9
OCH3
O
MeO
NH
N
H
O
4q
200
180
160
140
120
100
80
60
40
20
0
f1 (ppm)

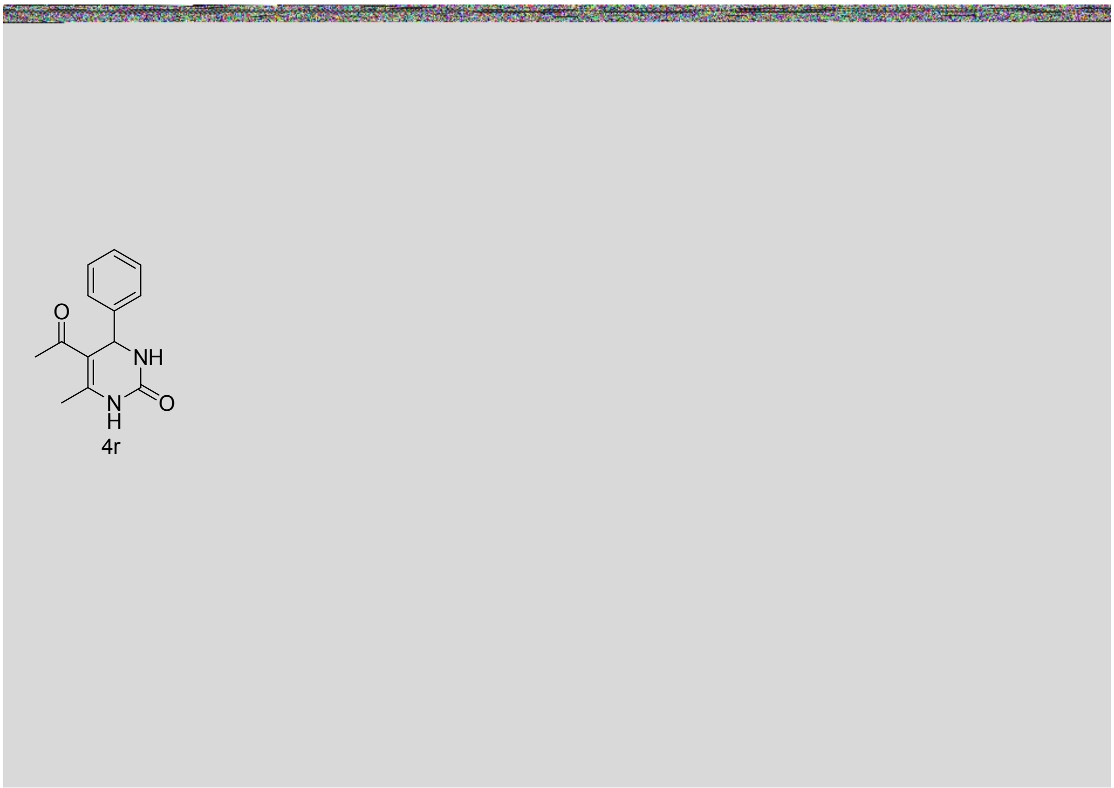
O
NH
N
H
O
4r

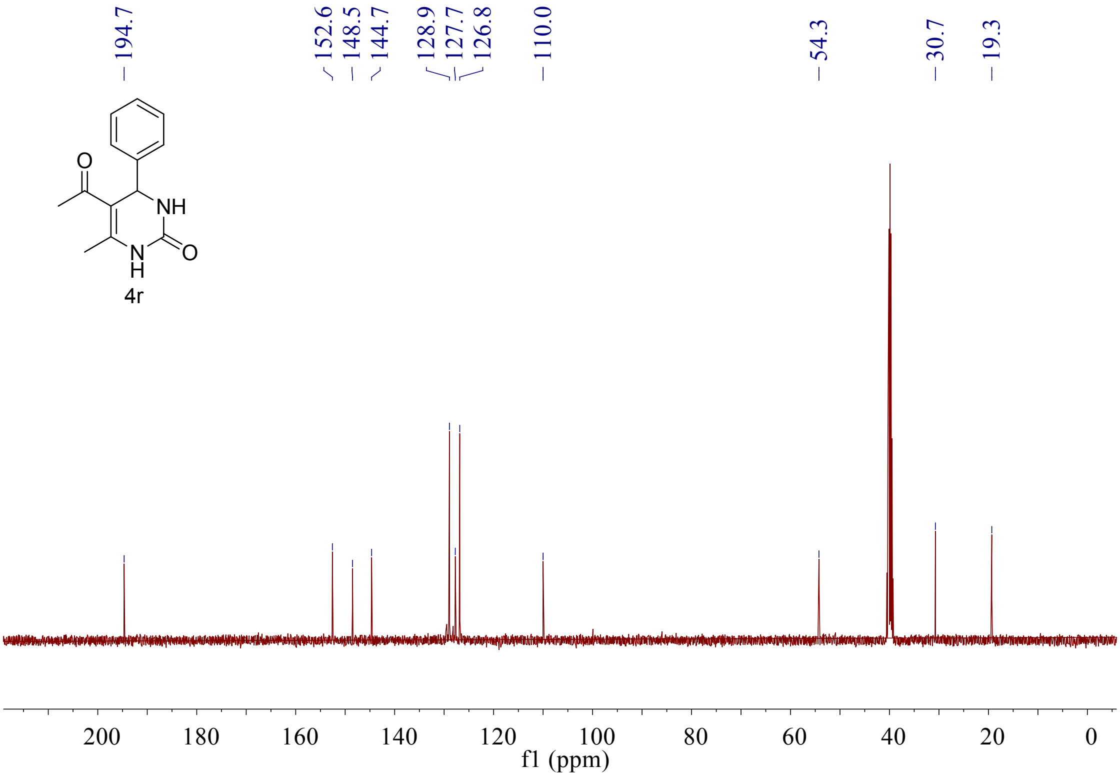
194.7
152.6
148.5
144.7
128.9
127.7
126.8
110.0
54.3
30.7
19.3
O
NH
N
H
O
4r
200
180
160
140
120
100
80
60
40
20
0
f1 (ppm)

**5. HR-ESI-MS studies for proposed mechanism.**

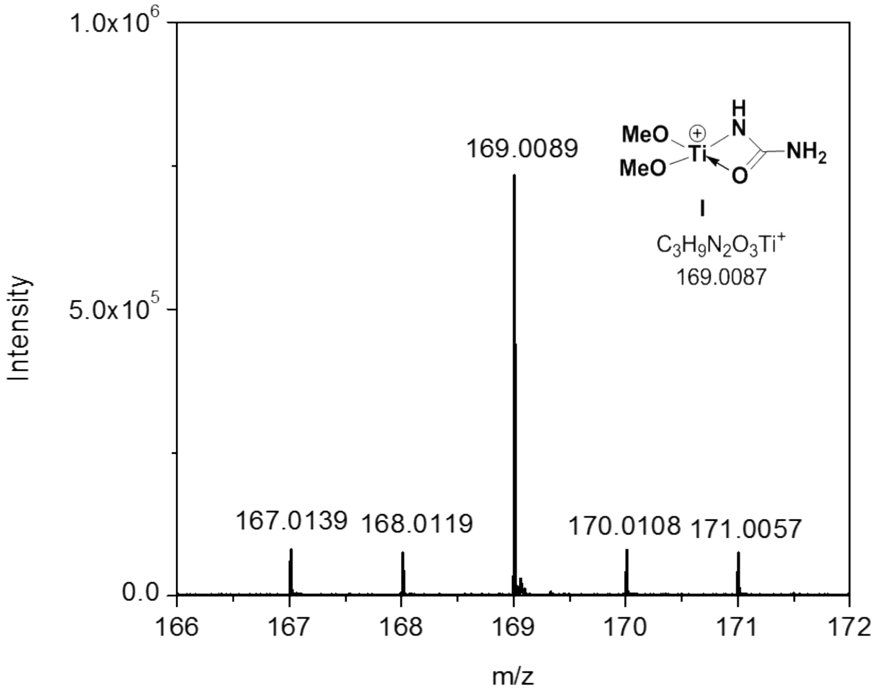


**Figure S5-1.** ESI(+)-MS spectra of intermediate I mode.

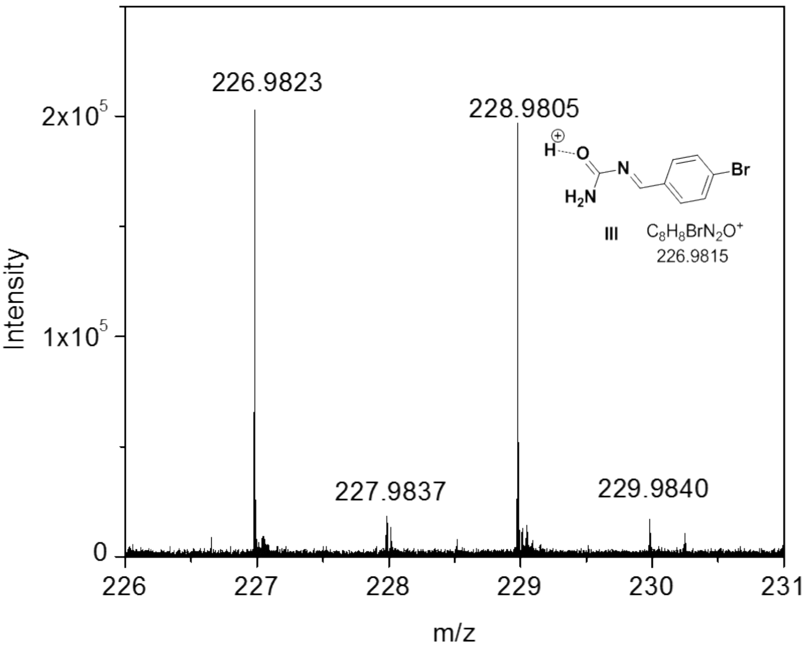

**Figure S5-2.** ESI(+)-MS spectra of intermediate III mode.

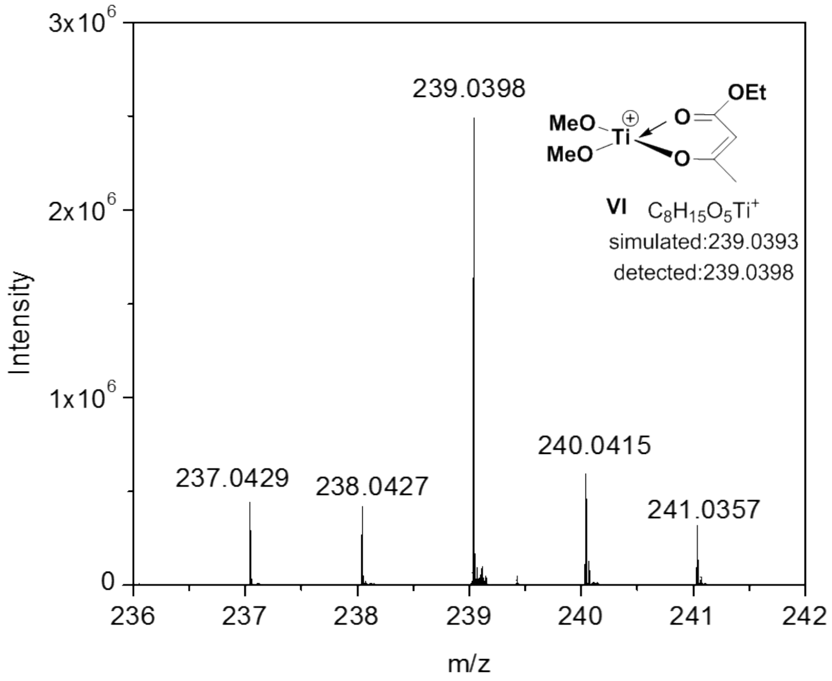


**Figure S5-3.** ESI(+)-MS spectra of intermediate VI mode.

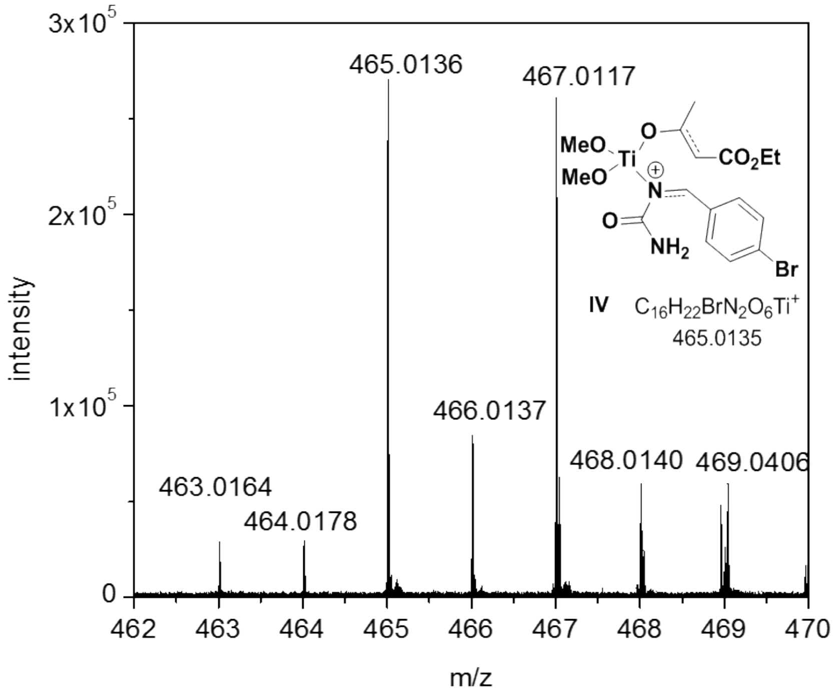


**Figure S5-4.** ESI(+)-MS spectra of intermediate IV mode.

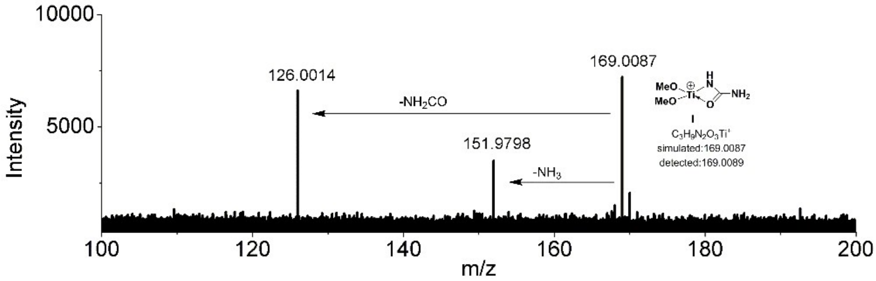


**Figure S5-5.** MS/MS spectrum and the fragment structures of intermediate I under positive ion mode.

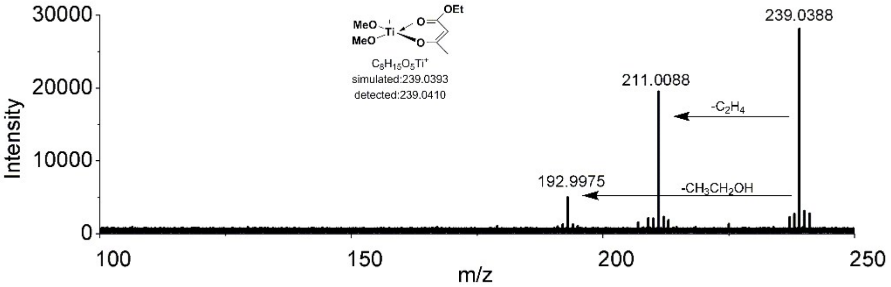


**Figure S5-6.** MS/MS spectrum and the fragment structures of intermediate VI under positive ion mode.

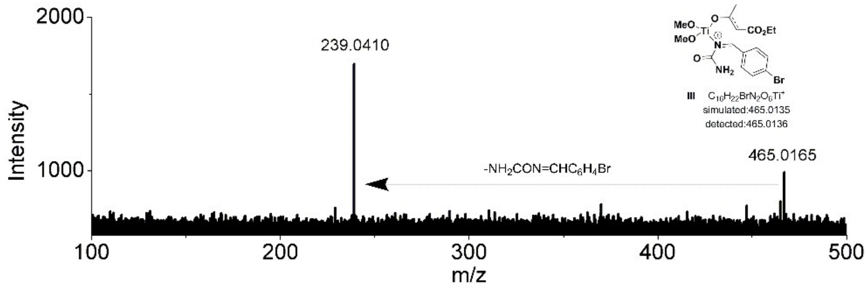


**Figure S5-7.** MS/MS spectrum and the fragment structures of intermediate III under positive ion mode.

## 6. $^{13}$C NMR Spectra studies for proposed mechanism

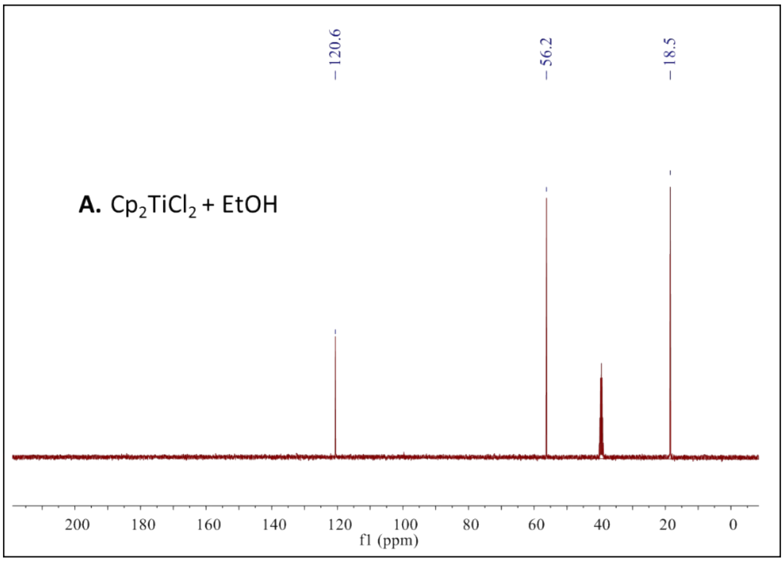


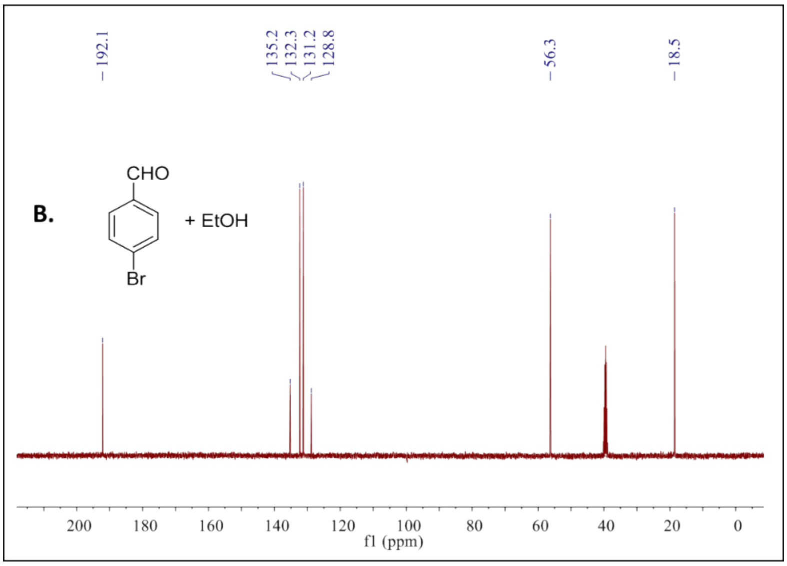


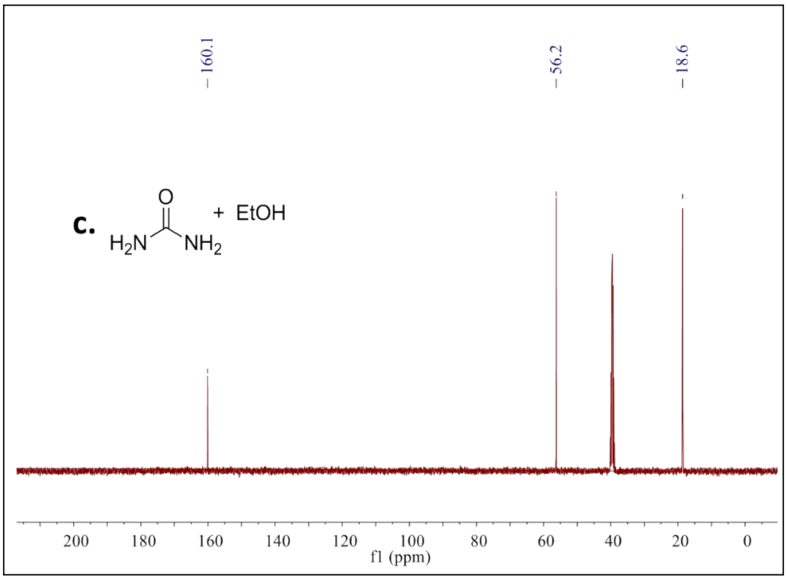

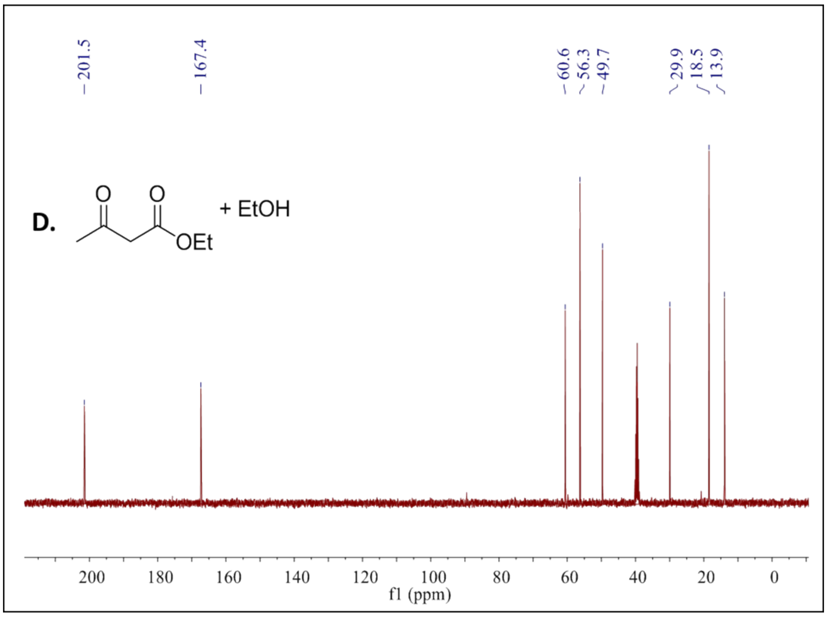
201.5
167.4
60.6
56.3
49.7
29.9
18.5
13.9
D.
+ EtOH
OEt
200
180
160
140
120
100
80
60
40
20
0
f1 (ppm)

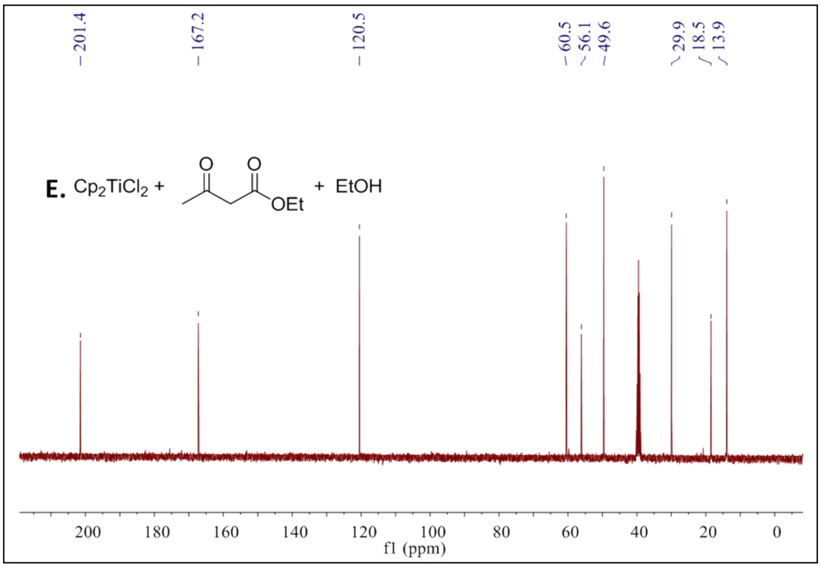
201.4
167.2
120.5
60.5
56.1
49.6
29.9
18.5
13.9
E.
$Cp_2TiCl_2$ +
+ EtOH
OEt
200
180
160
140
120
100
80
60
40
20
0
f1 (ppm)

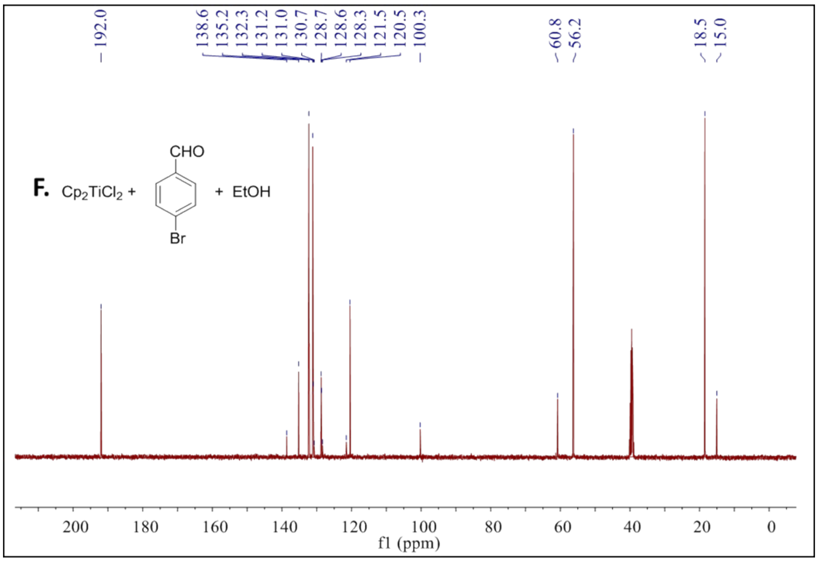
192.0
138.6
135.2
132.3
131.2
131.0
130.7
128.7
128.6
128.3
121.5
120.5
100.3
60.8
56.2
18.5
15.0
F.
$Cp_2TiCl_2$ +
CHO
Br
+ EtOH
200
180
160
140
120
100
80
60
40
20
0
f1 (ppm)

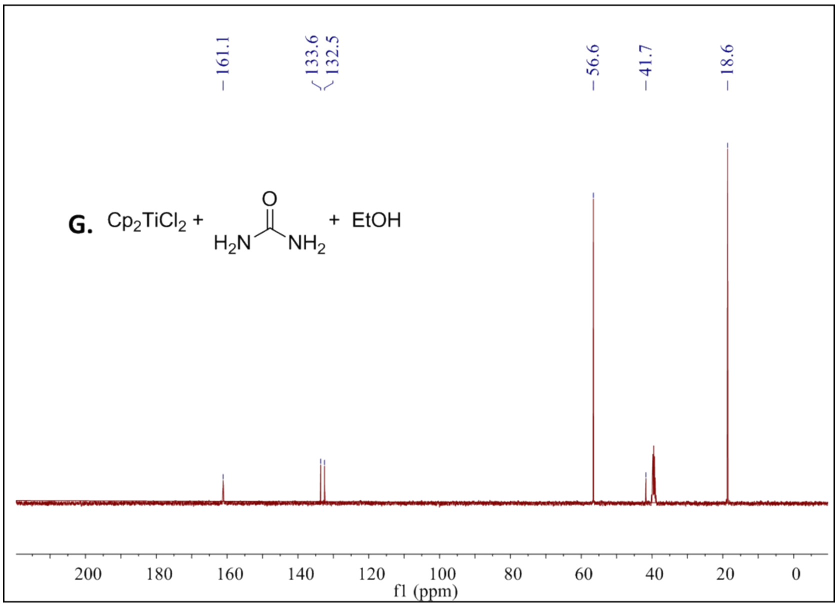
161.1
133.6
132.5
56.6
41.7
18.6
G. $Cp_2TiCl_2$ + O H2N NH2 + EtOH
200 180 160 140 120 100 80 60 40 20 0
f1 (ppm)

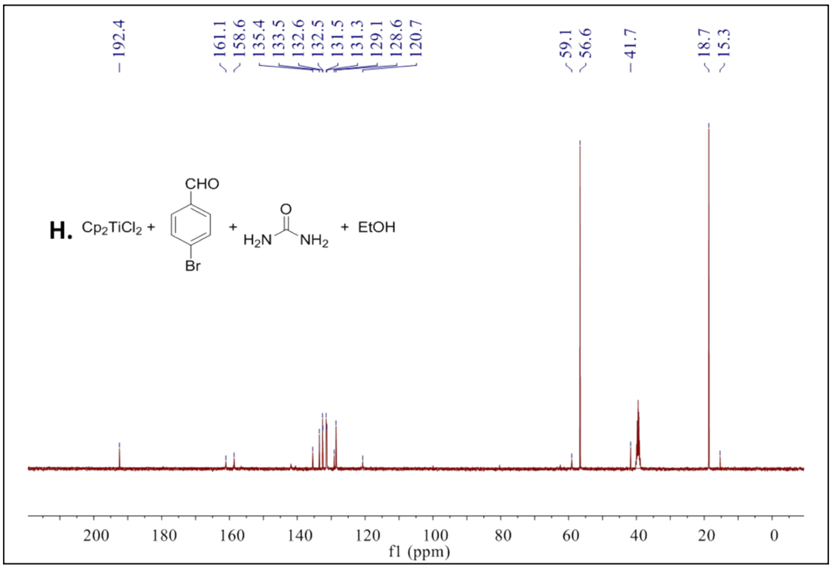
192.4
161.1
158.6
135.4
133.5
132.6
132.5
131.5
131.3
129.1
128.6
120.7
59.1
56.6
41.7
18.7
15.3
H. $Cp_2TiCl_2$ + CHO Br + O H2N NH2 + EtOH
200 180 160 140 120 100 80 60 40 20 0
f1 (ppm)

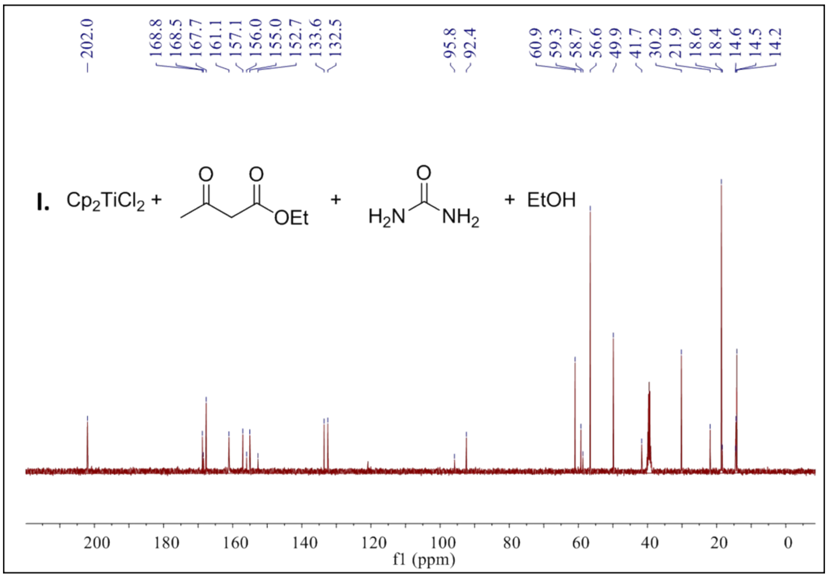
202.0
168.8
168.5
167.7
161.1
157.1
156.0
155.0
152.7
133.6
132.5
95.8
92.4
60.9
59.3
58.7
56.6
49.9
41.7
30.2
21.9
18.6
18.4
14.6
14.5
14.2
I. $Cp_2TiCl_2$ + O O OEt + O H2N NH2 + EtOH
200 180 160 140 120 100 80 60 40 20 0
f1 (ppm)
